# Preclinical platforms to study therapeutic efficacy of human γδ T cells

**DOI:** 10.1002/ctm2.814

**Published:** 2022-06-22

**Authors:** Lingling Ou, Huaishan Wang, Hui Huang, Zhiyan Zhou, Qiang Lin, Yeye Guo, Tara Mitchell, Alexander C. Huang, Giorgos Karakousis, Lynn Schuchter, Ravi Amaravadi, Wei Guo, Joseph Salvino, Meenhard Herlyn, Xiaowei Xu

**Affiliations:** ^1^ Department of Pathology and Laboratory Medicine University of Pennsylvania Philadelphia Pennsylvania USA; ^2^ Stomatological Hospital Southern Medical University Guangzhou China; ^3^ The First Affiliated Hospital of Jinan University Guangzhou China; ^4^ Department of Medicine University of Pennsylvania Philadelphia Pennsylvania USA; ^5^ Department of Surgery University of Pennsylvania Philadelphia Pennsylvania USA; ^6^ Department of Biology University of Pennsylvania Philadelphia Pennsylvania USA; ^7^ Molecular and Cellular Oncogenesis Program The Wistar Institute Philadelphia Pennsylvania USA

**Keywords:** epigenetic modifiers, immune checkpoint blockade, immunotherapy, melanoma 3D models, γδ T cells

## Abstract

**Background:**

Gamma delta (γδ) T lymphocytes are promising candidate for adoptive T cell therapy, however, their treatment efficacy is not satisfactory. Vδ2 T cells are unique to primates and few suitable models are available to assay their anti‐tumour function.

**Methods:**

We tested human γδ T cell activation, tumour infiltration, and tumour‐killing in four three‐dimensional (3D) models, including unicellular, bicellular and multicellular melanoma spheroids, and patient‐derived melanoma organoids. We studied the effects of checkpoint inhibitors on γδ T cells and performed a small molecule screen using these platforms.

**Results:**

γδ T cells rapidly responded to melanoma cells and infiltrated melanoma spheroids better than αβ T cells in PBMCs. Cancer‐associated fibroblasts (CAFs) in bicellular spheroids, stroma cells in multicellular melanoma spheroids and inhibitory immune cells in organoids significantly inhibited immune cell infiltrates including γδ T cells and lessened their cytotoxicity to tumour cells. Tumour‐infiltrating γδ T cells showed exhausted immunophenotypes with high checkpoints expression (CTLA‐4, PD‐1 and PD‐L1). Immune checkpoint inhibitors increased γδ T cell infiltration of 3D models and killing of melanoma cells in all four 3D models. Our small molecule screen assay and subsequent mechanistic studies demonstrated that epigenetic modifiers enhanced the chemotaxis and cytotoxicity of γδ T cells through upregulating MICA/B, inhibiting HDAC6/7 pathway and downregulating the levels of PD‐L1 and PD‐L2 in CAFs and tumour cells. These compounds increased CXCR4 and CD107a expression, IFN‐γ production and decreased PD‐1 expression of γδ T cells.

**Conclusions:**

Tumour‐infiltrating γδ T cells show exhausted immunophenotypes and limited anti‐tumour capacity in melanoma 3D models. Checkpoint inhibitors and epigenetic modifiers enhance anti‐tumour functions of γδ T cells. These four 3D models provided valuable preclinical platforms to test γδ T cell functions for immunotherapy.

## Graphical abstract

The immunosuppressive TME of these 3D models contained of tumour cells, CAFs and immune cells accompanied by high expression of PD‐L1, PD‐L2 and reduced expression of MICA/B. The immune checkpoint inhibitors and epigenetic modifiers enhance the efficacy of γδ T cells against 3D models. These four 3D models provided valuable preclinical platforms to test γδ T cell functions for immunotherapy.

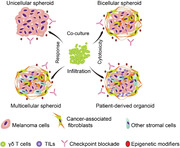

## 1 INTRODUCTION

γδ T cells possess both innate and adaptive immune cell traits.^1^ γδ T cell accumulation in cancer tissues is the best predictor for better prognosis among 22 different types of immune cells across 39 malignancies.^2^ Since γδ T cells recognise tumour cells without classical MHC restriction and co‐stimulatory molecules, γδ T cells are attractive candidates for allogeneic adoptive therapy in various cancers without inducing graft‐versus‐host disease.^3^, ^4^ γδ T cells exert excellent anti‐tumour activity in the two‐dimensional (2D) culture, however, the efficacy of adoptive γδ T cell therapy is variable. Both clinical trials and basic research are needed to explore strategies to overcome resistance to γδ T cell therapy.^5^

It appears that γδ T cell anti‐tumor function is significantly inhibited within the tumor microenvironment (TME).^6^, ^7^ The TME is comprised of malignant tumour cells, stroma and immune cells.^8^ Cancer‐associated fibroblasts (CAFs) are one of the most prominent cells in TME.^9^ CAFs secrete cytokines and extracellular matrix components to modify tumour architecture and enhance tumour proliferation and migration properties.^10^, ^11^ CAFs also interact with tumor infiltrating immune cells and inhibit αβ T cell infiltration and normal function.^12^ Thus, the TME play a crucial role in tumour progression and treatment resistance.

Vγ9Vδ2T cells, the most common γδ T cells used for adoptive immunotherapy, are unique to primates.^13^ Mouse models are not ideal to test efficacy of Vδ2 T cell‐based immunotherapy. Monolayer tumour cell culture cannot recapitulate the complex features of the TME in patients.^14^ Three‐dimensional (3D) culture, particularly patient‐derived tumor organoids^15^ mimic tumour tissue structure and may be used to study interaction of cancer cells with stromal cells and immune cells in vitro.^16^, ^17^ Multiple studies show that αβ T cells^18^ and NK cells^19^ are capable of infiltrating and killing tumour spheroids.^20^ However, few studies have examined how γδ T cells interact with melanoma cells in 3D models.

We therefore developed four different 3D models to study γδ T cell migration, infiltration, activation and cytotoxicity. The four different 3D models including unicellular melanoma spheroids, bicellular tumour‐fibroblast spheroids, multicellular patient‐derived spheroids and melanoma patient‐derived organoids (MPDOs). From unicellular spheroids to MPDOs, the complexity of the 3D models increases significantly with CAFs, other stromal cells and immune cells.

We demonstrated that γδ T cells rapidly migrated to and infiltrated tumour spheroids faster than αβ T cells. Inclusion of CAFs in the spheroids significantly inhibited γδ T cell infiltration. Tumour infiltrating γδ T cells expressed high level of checkpoint proteins in the 3D models. Anti‐PD‐1 and anti‐CTLA‐4 mAbs increased γδ T cell infiltration and killing of melanoma cells in all four models. In our small molecule screen assays, we discovered that Entinostat and Vorinostat enhanced γδ T cell tumour killing function. Mechanistically, these compounds enhanced the chemotaxis and cytotoxicity of γδ T cells through upregulating MICA/B and downregulating the levels of PD‐L1 and PD‐L2 on CAFs and tumour cells; while upregulating CXCR4, CD107a expression and IFN‐γ production in γδ T cells. 3D models provide useful preclinical platforms to study human γδ T cell functions for immunotherapy.

## 2 RESULTS

### 2.1 γδ T cells infiltrate unicellular melanoma spheroids more efficiently than αβ T cells

**FIGURE 1:**
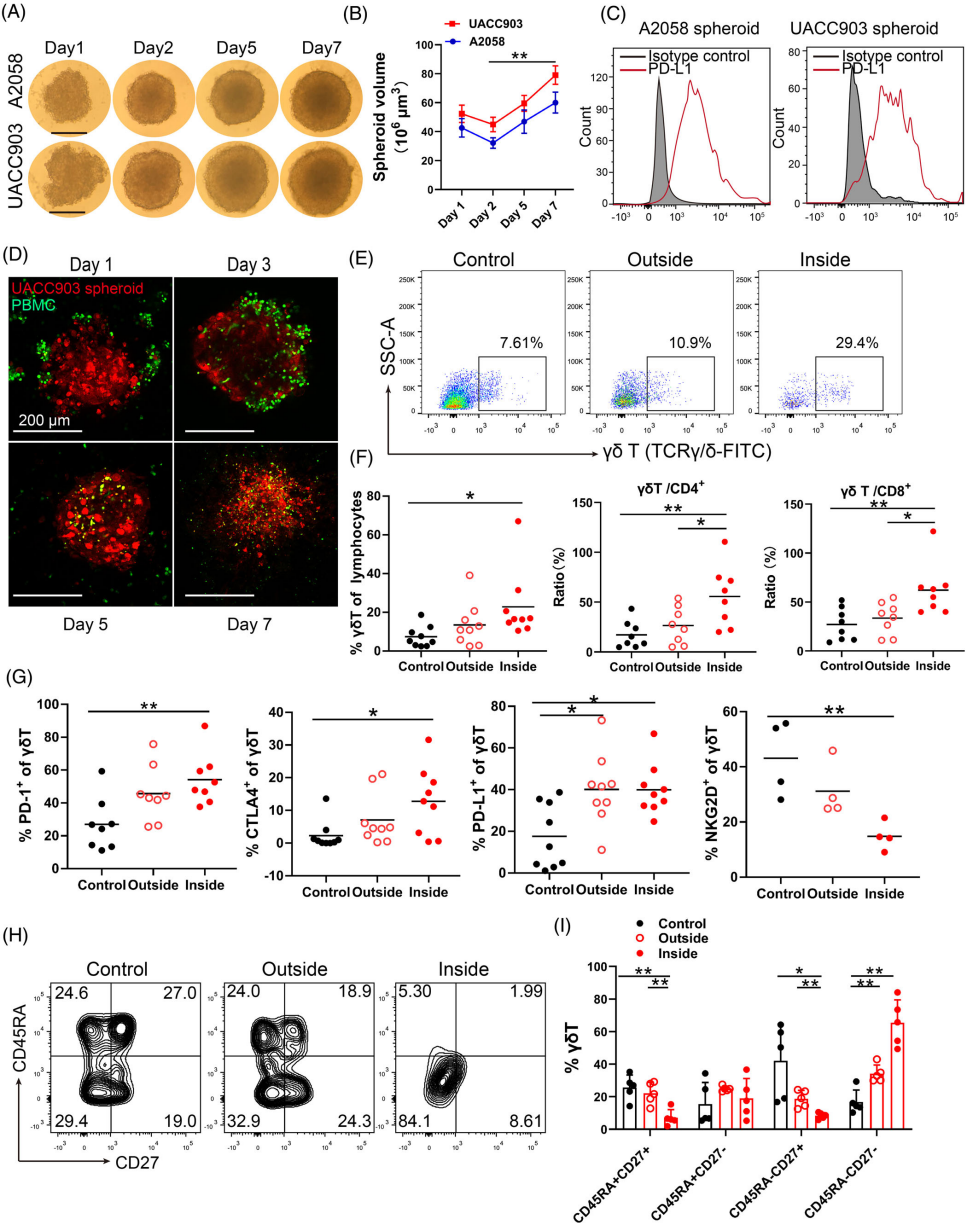
γδ T cells infiltrate unicellular melanoma spheroids. (A) A2058 and UACC903 unicellular melanoma spheroids. 2 × 10^4^ melanoma cells were seeded in each well and spheroid images were taken. Scale bar, 200 μm. (B) Time course of unicellular spheroid growth. Spheroid size was measured using a micrometer. ** *p* < 0.01. (C) PD‐L1 expression in unicellular A2058 and UACC903 spheroids. The tumour spheroids were disassociated and expression of PD‐L1 was measured by FACS. (D) Co‐culture of UACC903 spheroids and peripheral blood mononuclear cells (PBMCs) with time. 2 × 10^4^ melanoma cells were labelled with Cell‐tracker orange CMTMR (red), while 4 × 10^4^ PBMCs were labelled with CFSE (green). Representative images from days 1, 3, 5, and 7 were shown. Scale bar, 200 μm. (E) Representative scatter plots of γδ T cells in the medium (outside) or inside the spheroids at day 3 after co‐culture. γδ T cells were gating on CD3^+^ lymphocytes. Control wells do not contain tumor spheroids. (F) Percentage of γδ T cells in lymphocytes, ratio of γδ T cells/CD4^+^ cells and γδ T cells/CD8^+^ cells at day 3 after co‐culture were showed. γδ T, CD4 T and CD8 T cells in the medium (outside) or inside the spheroids were measured by FACS. Control wells do not contain tumor spheroids. Each dot in this panel represents a unique donor. * *p* < 0.05; ** *p* < 0.01. (G) CTLA‐4, PD‐1, PD‐L1 and NKG2D expression in γδ T cells in the medium (outside) or inside the spheroids. Control wells did not contain tumour spheroids. Each dot in this panel represents a unique donor. * *p* < 0.05; ** *p* < 0.01. (H) The phenotypic subsets of γδ T cells outside or inside of the spheroids were shown in contour plots. Control wells did not contain tumour spheroids. (I) The phenotypic subsets of γδ T cell distributed in effector memory (EM, CD27‐CD45RA‐), terminal‐differentiated effector memory (TDEM, CD27‐CD45RA+), naive (N, CD27+CD45RA+), and central memory (CM, CD27+CD45RA‐) subsets. Control wells did not contain tumour spheroids. * *p* < 0.05; ** *p* < 0.01

A2058 and UACC903 unicellular melanoma spheroids were generated by seeding tumour cells on agarose‐coated 96‐well plates. Melanoma cells aggregated and started to form spheroids at day 1 and well‐formed spheroids were seen at day 2; the spheroid size grew over 500–600 μm at day 7; and the shape of A2058 and UACC903 spheroids were similar (Figure 1A,B). Melanoma cells in the spheroids expressed high level of PD‐L1 measured by FACS (Figure 1C).

To study T cell infiltration, spheroids (labelled with Cell‐Tracker Orange CMTMR, red) were co‐cultured with peripheral blood mononuclear cells (PBMCs, labelled with carboxyfluorescein succinimidyl ester (CFSE), green) from healthy donors. Immune cells were initially present on the surface of the spheroids at day 1, many immune cells infiltrated the spheroids at day 3, the immune cells persisted in the spheroids at day 5 and 7, and the spheroids were destroyed gradually (Figure 1D). The proportion of γδ T cells was significantly higher inside of the spheroids than that outside of spheroids (in the medium) or in control wells (without spheroids) at day 3 (Figure 1E). In contrast, there was no significant change of CD8^+^ T and CD4^+^ T cells in the spheroids comparing that outside of the spheroids or in control wells (without spheroids, Figure S1A,B). The ratios of γδ T/CD4^+^ and γδ T/CD8^+^ T cells were significantly increased in the spheroids compared to those outside of the spheroids (Figure 1F), supporting that γδ T cells proliferate and infiltrate tumour spheroids more efficiently than CD4^+^ and CD8^+^ T cells.

The infiltrating γδ T cells displayed increased expression of CTLA‐4, PD‐1 and PD‐L1 compared to those in the control wells (γδ T cells without spheroids, Figure 1G and Figure S1C). Similar findings were observed in spheroid infiltrating CD8^+^ and CD4^+^ T cells (Figure S1D,E). A decreased expression of NKG2D was also observed in γδ T cells, indicating an engagement of NKG2D in the spheroids.

γδ T cells are comprised of varying proportions of effector memory (CD27–CD45RA–), central memory (CD27+CD45RA–), terminal‐differentiated effector memory (CD27–CD45RA+) and naive T cells (CD27+CD45RA+) in different physiological conditions.^21^, ^22^ The infiltrating γδ T cells showed increased percentages of effector memory cells (EM, CD27–CD45RA–) compared to cells outside of spheroids (Figure 1H,I). These results support that the effector memory γδ T cells are apt to infiltrate the spheroids. Alternatively, the γδ T cells may differentiate into effector memory subset as they infiltrate the spheroids. These results suggest that γδ T cells are one of the fastest responding immune cells when encountering with tumour cells.

### 2.2 CAFs increase immune suppression in the spheroids

**FIGURE 2:**
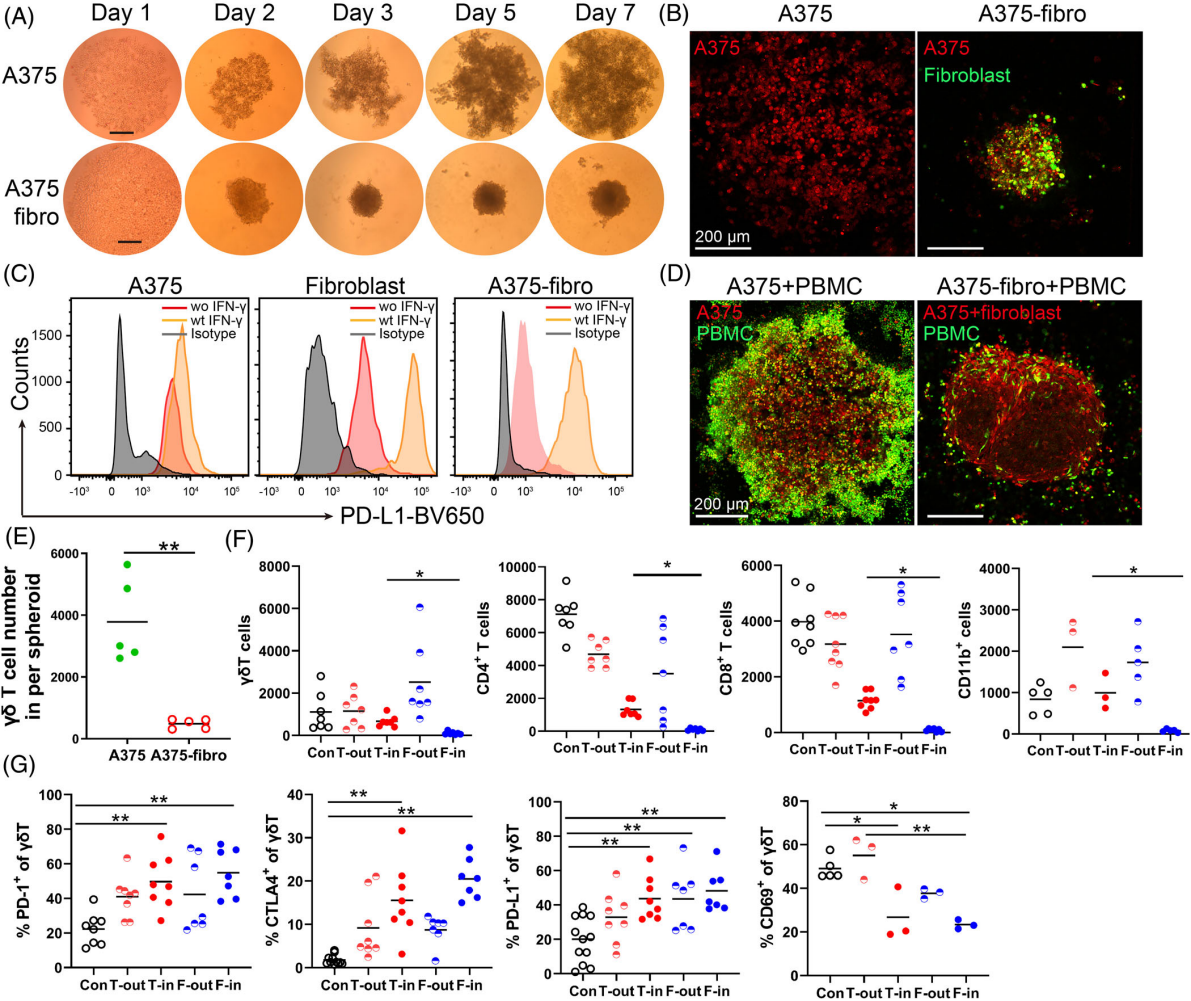
Cancer‐associated fibroblasts inhibit immune cell infiltrate. (A) A375 cell cluster and A375‐fibroblast bicellular spheroids. Melanoma cells and fibroblasts were mixed at a 1:2 ratio and seeded on agarose. Representative images of A375 cell cluster and A375‐fibroblast spheroids at days 1, 3, 5 and 7 were shown. Scale bar, 250 μm. (B) Representative fluorescent images of A375 cell cluster and A375‐fibroblast spheroid. Tumour cells in the spheroids were labelled with CellTracker™ Orange CMRA dye (red), and the fibroblast cells were labelled with CFSE (green). Scale bar, 200 μm. (C) PD‐L1 expression in response to IFN‐γ on A375 cells, fibroblasts and CAFs in A375‐fibroblast spheroids. A375, fibroblasts and A375‐fibroblast spheroids were cultured with 50 ng/ml IFN‐γ for 24 h and PD‐L1 expression on A375, fibroblasts and CAFs was measured by FACS. (D) Representative images of A375 cell cluster and A375‐fibroblast spheroids co‐cultured with peripheral blood mononuclear cells (PBMCs). Melanoma cells and fibroblasts were labelled with orange CMRA dye (red), and PBMCs were labelled with CFSE (green). Scale bar, 200 μm. (E) Infiltrating γδ T cells in the A375 and A375‐fibroblast spheroids were measured by FACS. Spheroids were co‐cultured with PBMCs for 24 h and CFSE labelled immune cells were quantified by FACS. *n* = 5; **p < 0.01. (F) Average γδ T cells, CD4^+^ T cells, CD8^+^ T cells and CD11b^+^ cells outside or inside of an A2058 tumour spheroid (T‐out or T‐in) and A2058‐fibroblast spheroid (F‐out or F‐in) were measured by FACS. * *p* < 0.05. (G) The CTLA‐4, PD‐1, PD‐L1 and CD69 expression on γδ T cells outside or inside of A2058 tumour spheroid (T‐out or T‐in) and A2058‐fibroblast spheroids (F‐out or F‐in). * *p* < 0.05. **p < 0.01. *n* = 3–7. Con: PBMCs cultured without tumour spheroids, T‐out: outside of unicellular tumour spheroids, T‐in: inside of unicellular tumour spheroids, F‐out: outside of A2058‐fibroblast spheroids, F‐in: inside of A2058‐fibroblast spheroids

Fibroblasts play a pivotal role in the TME and rigid extracellular matrix impairs T cell infiltration.^23^, ^24^ We found that not all melanoma cell lines form spheroids on the agarose. A375 melanoma cells were not able to form spheroids on the agarose, however, a mixture of A375 cells and fibroblasts was able to form tight spheroids and their size increased slowly over time (Figure 2A). When fibroblasts (GM01948 cells) were introduced into the spheroids, the bicellular melanoma–fibroblast spheroids (A375‐fibro and A2058‐fibro) were smaller than unicellular tumour spheroids (Figure 2B and Figure S2A). Fibroblast markers, α‐smooth muscle actin (α‐SMA) and fibroblast activation protein (FAP) were highly expressed in the A2058‐fibroblast spheroids (Figure S2B). These CAFs expressed high level of PD‐L1 after stimulation with 50 ng/ml IFN‐γ for 24 h (Figure 2C).

After co‐culturing with PBMCs for 24 h, the bicellular spheroids recruited significantly fewer infiltrating immune cells compared to unicellular melanoma spheroids (Figure 2D, E and Figure S2C). Significantly fewer γδ T cells, CD4^+^, CD8^+^ T or CD11b^+^ cells were in the melanoma–fibroblast bicellular spheroids than those in the unicellular spheroids (Figure 2F). Infiltrating γδ T in bicellular spheroids expressed significantly higher level of CTLA‐4, PD‐1, PD‐L1 and lower level of CD69 compared to those of controls; while comparable levels of CTLA‐4, PD‐1, PD‐L1 and CD69 as those in unicellular spheroids (Figure 2G). Similar results were observed on PD‐1 expression level in spheroid infiltrating CD4^+^ T and CD8^+^ T cells, and on PD‐L1 expression and TGF‐β production in spheroid infiltrating CD11b^+^ cells (Figure S2E). Similar to the phenotypic changes in the unicellular melanoma spheroids, more effector memory γδ T cells were present in the bicellular spheroids compared with that outside of the spheroids (Figure S2F).

### 2.3 Immune Checkpoint blockade (ICB) enhances melanoma killing and infiltration of γδ T cells in melanoma spheroids

**FIGURE 3:**
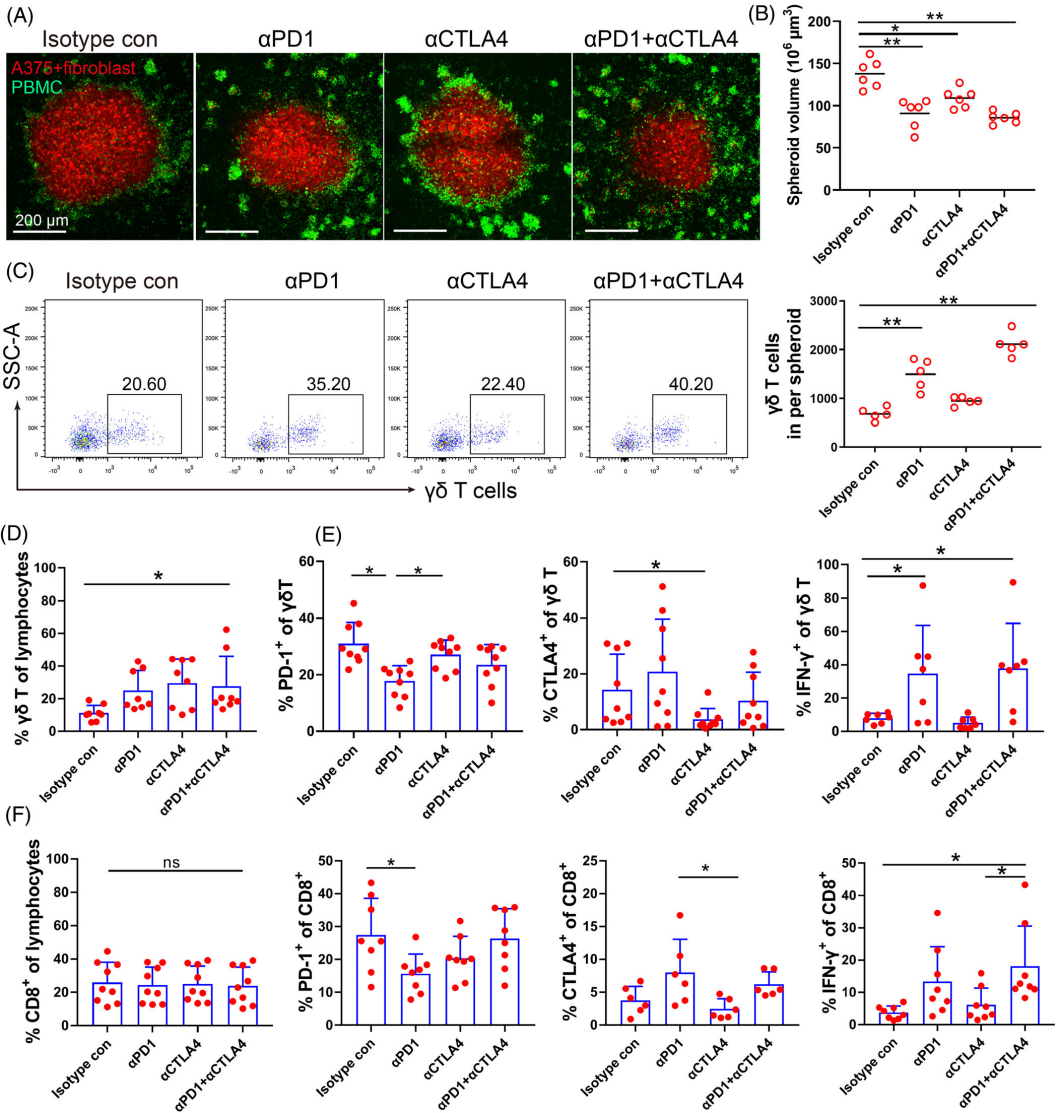
Anti‐PD‐1 and anti‐CTLA‐4 mAbs enhance T cell infiltration. (A) Representative images of A375‐fibroblast spheroids co‐cultured with peripheral blood mononuclear cell (PBMC) and treated by ICB, the spheroid cells were labelled with oorange dye (red), and PBMCs were labelled with CFSE (green). A375‐fibroblast spheroids were co‐cultured with PBMCs in the presence of anti‐PD‐1 (Pembrolizumab, 50 μg/ml) and/or anti‐CTLA‐4 (Ipilimumab, 50 μg/ml) mAbs for 48 h. Scale bar, 200 μm. (B) Spheroid volume was measured after treatment for 48 h. *n* = 6, *p < 0.05. ** *p* < 0.01. (C) Representative scatter plots and the absolute number of γδ T cells inside of the A375‐fibroblast spheroids after treatment. γδ T cells were gated on CD3^+^ lymphocytes. **p < 0.01. (D) Proportion of γδ T cells in the A375‐fibroblast spheroids was measured after treatment. *p < 0.05. (E) PD‐1, CTLA‐4 expression and IFN‐γ production in tumour infiltrating γδ T cells was measured by FACS after treatment. *p < 0.05. (F) Proportion of CD8^+^ T cells, CTLA‐4and PD‐1 expression, and IFN‐γ production in tumour infiltrating CD8^+^ T cells were measured by FACS after treatment. *p < 0.05; ns, no significant difference

PBMC co‐cultured with tumour spheroids simulates immune cells in blood or in tissues reacting with tumours in vivo.^25^, ^26^ We assessed whether anti‐CTLA‐4 or anti‐PD‐1 mAbs alone or in combination increase the treatment efficacy of T cells in PBMC against melanoma–fibroblast bicellular spheroids. A375‐fibroblast bicellular spheroids were co‐cultured with PBMC in the presence of ICB for 48 h. The anti‐CTLA‐4 or anti‐PD‐1 mAbs or their combination significantly decreased the size of spheroids compared to isotype control (Figure 3A,B); anti‐PD1 mAbs and the combination significantly increased infiltrating γδ T cells in the spheroids (Figure 3C,D). Despite anti‐CTLA‐4/ PD‐1 mAbs reduced the size of spheroids, the 3D structure of spheroids was not completely destroyed.

Anti‐PD‐1 mAbs decreased PD‐1 expression, while anti‐CTLA‐4 mAbs reduced CTLA‐4 level in infiltrating γδ T cells. Anti‐PD‐1 mAbs or the anti‐CTLA‐4/ PD‐1 combination increased IFN‐γ production of γδ T cells (Figure 3E). Similar effects were observed on tumour infiltrating CD8^+^ T cells (Figure 3F). The efficacy of ICB on CD4^+^ T cells was less prominent than on CD8^+^ T cells (Figure S3A). Anti‐CTLA‐4/PD‐1 or the combination significantly decreased CD11b^+^ and CD14^+^ cells in the spheroids, and the combination decreased the expression of PD‐L1 and TGF‐β production in CD11b^+^ macrophages, and PD‐1 and PD‐L1 levels in CD14^+^ myeloid cells (Figure S3B, S3C). These results support that ICB not only enhances the function of CD8^+^ T cells, but also improves the infiltration and cytotoxicity of γδ T cells.

### 2.4 Expanded γδ T cells show moderate anti‐tumor effects to melanoma spheroids

**FIGURE 4:**
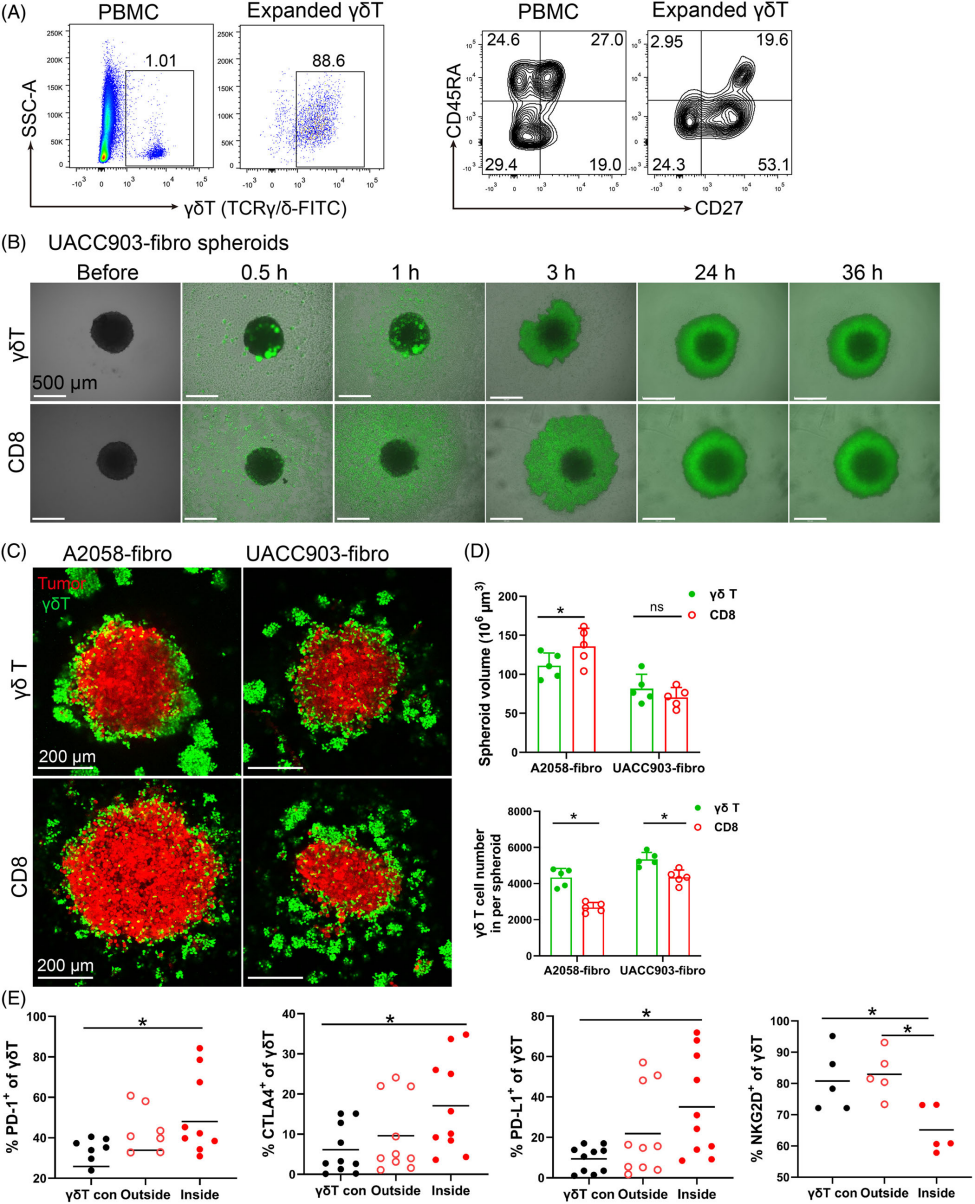
Efficacy of γδ T cell treatment in bicellular melanoma spheroids. (A) γδ T cell expansion and phenotypic subset distribution. γδ T cells were expanded from PBMCs for 10–13 days using 5 μM ZOL and 200 IU/ml IL‐2. Representative scatter plots showing expansion of γδ T cells and their phenotypic changes. (B) Time course of UACC903‐fibro spheroids co‐cultured with expanded γδ T cells and CD8 T cells were taken pictures by widefield microscopy. Scale bar, 500 μm. (C) Representative images of A2058‐fibro and UACC903‐fibro spheroid co‐culture with expanded γδ T cells or CD8^+^ T cells. Spheroid cells were labelled with orange dye (red), γδ T cells and CD8^+^ T cells were labelled with CFSE (green). Scale bar, 200 μm. (D) The size of A2058‐fibro and UACC903‐fibro spheroid and the infiltrating cell number was analysed after co‐culture with expanded γδ T cells or CD8^+^ T cells. *n* = 5; *p < 0.05; ns, no significant difference. (E) Proportion of γδ T cells and their CTLA‐4, PD‐1, PD‐L1 and NKG2D expression on γδ T cells in the medium (outside) or inside of A2058‐fibroblast spheroids were measured by flow cytometry. γδ T con: expanded γδ T cells without spheroids. *n* = 5–10; *p < 0.05. The γδ T cells and CD8^+^ T cells were from the same donors

Adoptive immunotherapy of CD8^+^ T and γδ T cells have been tested in clinical trials.^27^, ^28^ To compare the chemotaxis and cytotoxicity of γδ T cells with CD8^+^ T cells from the same donor, we expanded and activated CD8^+^ T cells as previously described.^29^, ^30^ γδ T cells can be rapidly expanded from PBMCs and expanded γδ T cells have been used in autologous or allogenic adoptive therapies.^31^, ^32^ We expanded γδ T cells from PBMCs using Zoledronic acid (5 μM) and IL‐2 (200 IU/ml) as we previously described^33^ and the purity of γδ T cells reached approximately 90% after 10 days (Figure 4A). The expanded γδ T cells comprised of more effector memory and central memory T cells compared to those in PBMCs (Figure 4A).

We observed that the expanded γδ T cells migrated to tumor spheroids more rapidly than CD8^+^ T cells when co‐cultured with melanoma–fibroblast bicellular spheroids (Figure 4B and , Figure S4). The expanded γδ T cells and CD8^+^ T cells infiltrated melanoma–fibroblast bicellular spheroids and disrupted the three‐dimensional structure modestly after 36 h co‐culture (Figure 4C). Spheroid size was significantly smaller in γδ T‐treated A2058‐fibro spheroids than CD8^+^ T‐treated A2058‐fibro spheroids. However, no significant difference was observed in UACC‐903‐fibro spheroids, suggesting that the intrinsic characteristics of tumour cells may play a role in response to γδ T cells. More γδ T cells infiltrated the bicellular spheroids compared to CD8^+^ T cells (Figure 4D). The infiltrating γδ T cells displayed significantly increased expression of CTLA‐4, PD‐1 and PD‐L1, with decreased expression of NKG2D compared to controls (Figure 4E). These data support that expanded γδ T cells can infiltrate into melanoma spheroids, but they show exhausted immunophenotypes with increased expression of checkpoint proteins. The exhausted immunophenotypes of tumour infiltrating γδ T cells likely contribute to limited clinical efficacy of the γδ T cell‐based therapies.

### 2.5 Patient‐derived multicellular spheroids and melanoma patient‐derived organoids mimic the TME in melanoma

**FIGURE 5:**
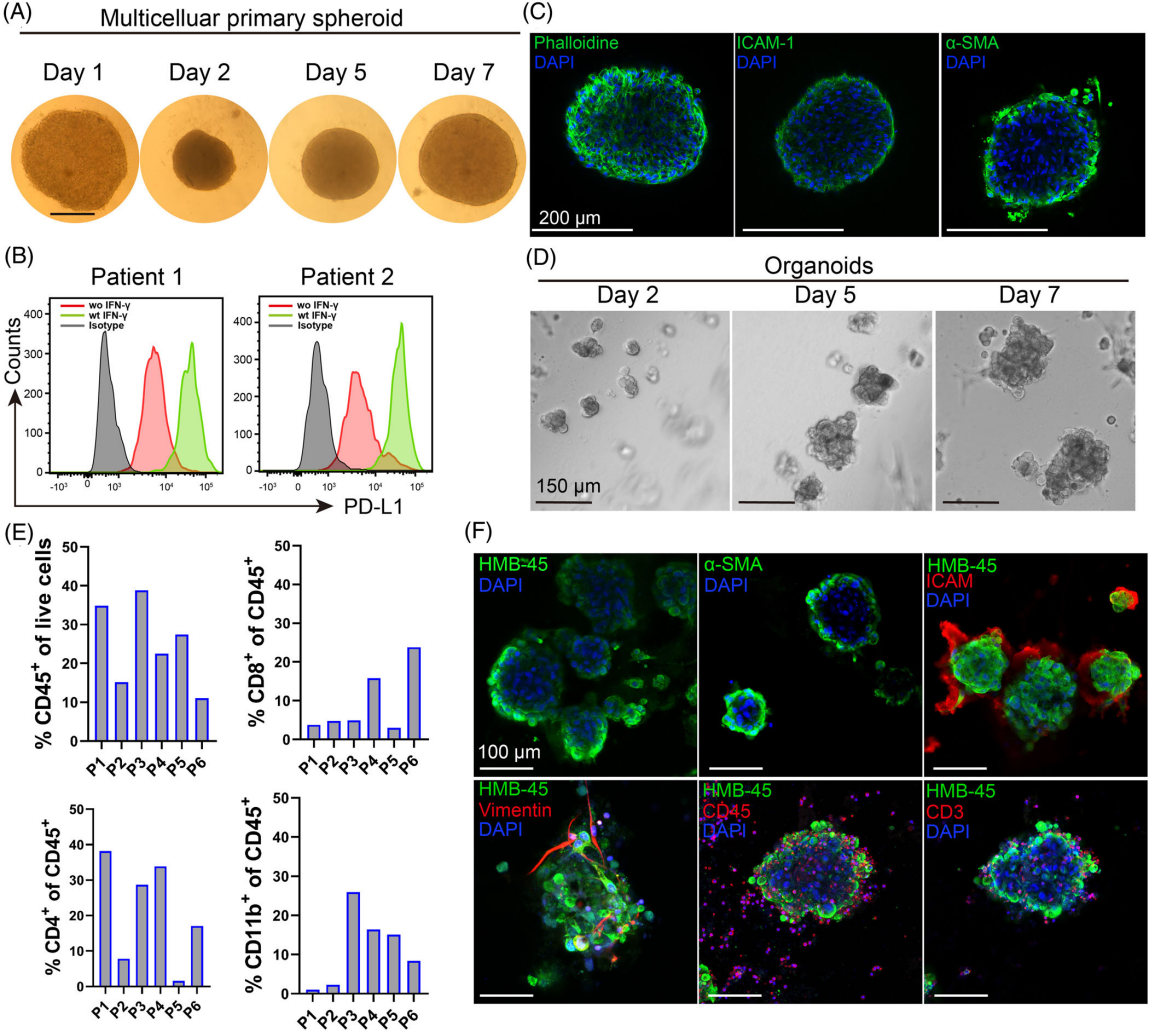
Patient‐derived multicellular spheroids and melanoma patient‐derived organoids (MPDOs). (A) Time course of patient‐derived multicellular spheroid growth. The adherent cultured cells from disassociated fresh patient melanoma tissues were digested and seeded on the agarose for 48 h to form multicellular spheroids. Images were taken by microscopy. Scale bar, 250 μm. (B) PD‐L1 expression in patient‐derived multicellular spheroid (patient 1 and patient 2) with or without IFN‐γ stimulation (50 ng/ml) was measured by FACS after spheroids were disassociated into single cells. (C) Immunofluorescent staining of Phalloidine, ICAM‐1 and α‐SMA in patient‐derived multicellular spheroid. Scale bar, 200 μm. (D) Representative images of MPDOs grew on the matrigel over time. Images were taken at days 2, 5, and 7. Single cells derived from fresh patient melanoma tissues were seeded onto the matrigel and cultured. Scale bar, 150 μm. (E) Cellular components of CD45^+^, CD8^+^, CD4^+^ and CD11b^+^ cells in the MPDOs were measured by flow cytometry. P1–P6, patient 1 to patient 6. (F) Immunofluorescent staining of HMB‐45, α‐SMA, ICAM‐1, Vimentin, CD45^+^ and CD3^+^ T cells in MPDOs. Scale bar, 100 μm

Although melanoma‐fibroblast bicellular spheroids contained CAFs, they lack many other cell types seen in the TME in vivo. To further mimic the TME in melanoma, we generated patient‐derived multicellular spheroids and MPDOs. Patient‐derived multicellular spheroids contained primary tumour cells, CAFs and other mesenchymal cells, but not immune cells. The cellular mixture was harvested from disassociated fresh melanoma tissues after adherent culture for 14–21 days, immune cells were gradually depleted during the culture period. The cellular mixture was seeded on the agarose for 48 h to form the melanoma multicellular spheroids. The patient‐derived multicellular spheroids initially contracted on the agarose on day 1, enlarged gradually and the central necrosis emerged when the size of spheroid reached over 800–1000 μm (Figure 5A and Figure S5). PD‐L1 was highly expressed on cells in the patient‐derived multicellular spheroids and IFN‐γ increased PD‐L1 expression in these cells (Figure 5B). We observed that cytoskeleton (phalloidine), fibroblasts (α‐SMA) and intercellular adhesion molecules‐I (ICAM‐I) were present in the multicellular spheroids (Figure 5C).

In contrast to patient‐derived multiple cellular spheroids, MPDOs also contain immune cells. Disassociated single‐cell suspension from fresh melanoma tissues was seeded onto the matrigel, and the organoids grew over time and the size reached about 200–300 μm at day 7 (Figure 5D). The main cellular components in the organoids were measured by FACS and immunohistochemistry. Immune cells including CD45^+^, CD8^+^, CD4^+^ and CD11b^+^ cells were present in the MPDOs (Figure 5E). Melanoma cells (HMB‐45), fibroblast (α‐SMA), ICAM‐I, vimentin and immune cells (CD45^+^ and CD3^+^ T cells) presented in the MPDOs by immunocytochemistry (Figure 5F).

### 2.6 Checkpoint blockade enhances anti‐tumor effect of expanded γδ T cells

**FIGURE 6:**
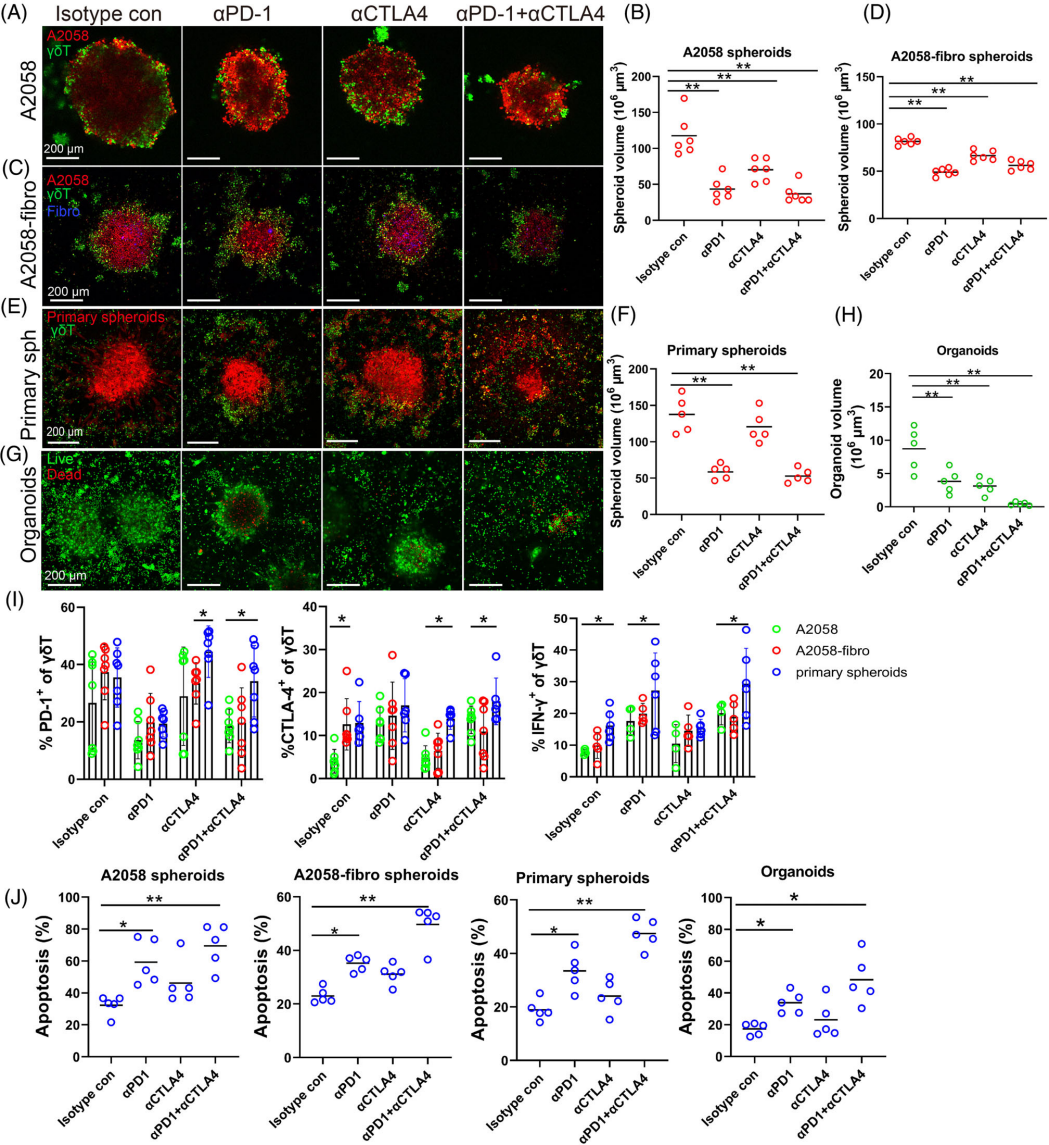
Anti‐PD‐1 and anti‐CTLA‐4 mAbs enhance γδ T cell treatment efficacy. (A) Representative images of A2058 spheroids co‐cultured with expanded γδ T cells and treated with 50 μg/ml anti‐PD‐1, 50 μg/ml anti‐CTLA‐4 or the combination of both mAbs for 48 h. Spheroid cells were labelled with orange dye (red) and γδ T cells were labelled with CFSE (green). Scale bar, 200 μm. (B) Volume of A2058 spheroids was measured after ICB treatment. *n* = 6, ** *p* < 0.01. (C). The images of A2058‐fibro spheroids co‐cultured with expanded γδ T cells and treated with anti‐CTLA‐4, anti‐PD‐1 or the combination. Spheroid cells were labelled with orange dye (red), fibroblast cells were stained with violet dye (blue) and γδ T cells were labelled with CFSE (green). Scale bar, 200 μm. (D) Volume of A2058‐fibro spheroids was measured after ICB treatment. *n* = 6, ** *p* < 0.01. (E) The images of multicellular patient‐derived primary spheroids co‐cultured with expanded γδ T cells and treated with ICB for 48 h. Scale bar, 200 μm. (F) Volume of patient‐derived primary spheroids was measured after ICB treatment. *n* = 5, **p < 0.01. (G) The AOPI staining image of melanoma patient‐derived organoids (MPDOs) co‐cultured with expanded γδ T cells combined with ICB treatment. Scale bar, 200 μm. (H) Volume of MPDOs was measured after ICB treatment. *n* = 5, **p < 0.01. (I) The expression of CTLA‐4 and PD‐1, and production of IFN‐γ in γδ T cells after ICB treatment in three types of spheroids were measured by FACS. *p < 0.05. (J) Statistical analysis of apoptosis in four different 3D models after combination therapy using expanded γδ T cells and ICB. *n* = 5. *p < 0.05; **p < 0.01

To enhance the treatment efficacy of γδ T cell therapy, we combined expanded Vδ2 T cells with ICB in A2058 unicellular spheroids (Figure 6A,B), A2058‐fibro bicellular spheroids (Figure 6C,D), multicellular spheroids (Figure 6E,F) and MPDOs (Figure 6G,H). Anti‐PD‐1 mAbs and the anti‐CTLA4/PD‐1 mAbs combination significantly enhanced the effect of Vδ2 T cells in all four models, while anti‐CTLA‐4 mAbs did not have significant effect. Similar results were observed in UACC903 spheroids (Figure S6A,B), A375‐fibro spheroids (Figure S6C,D) and MPDOs from another patients (Figure S6E).

We found that the expression levels of CTLA‐4 and PD‐1 in the spheroid infiltrating γδ T cells were variable in the unicellular, bicellular and multicellular spheroids (Figure 6I). Anti‐PD‐1 mAbs decreased PD‐1 expression, anti‐CTLA‐4 mAbs reduced CTLA‐4 expression in γδ T cells in all the three models. Anti‐PD‐1 mAbs and the combination enhanced IFN‐γ production of γδ T cells in all the three models (Figure 6I). γδ T cells in combination with anti‐PD‐1 mAbs or in combination with anti‐ CTLA‐4/PD‐1 induced more cell apoptosis than that control γδ T cells, while anti‐CTLA‐4 mAbs had no significant effect (Figure 6J).

### 2.7 Small molecule compound screen to discover compounds that enhance γδ T cell function

**FIGURE 7:**
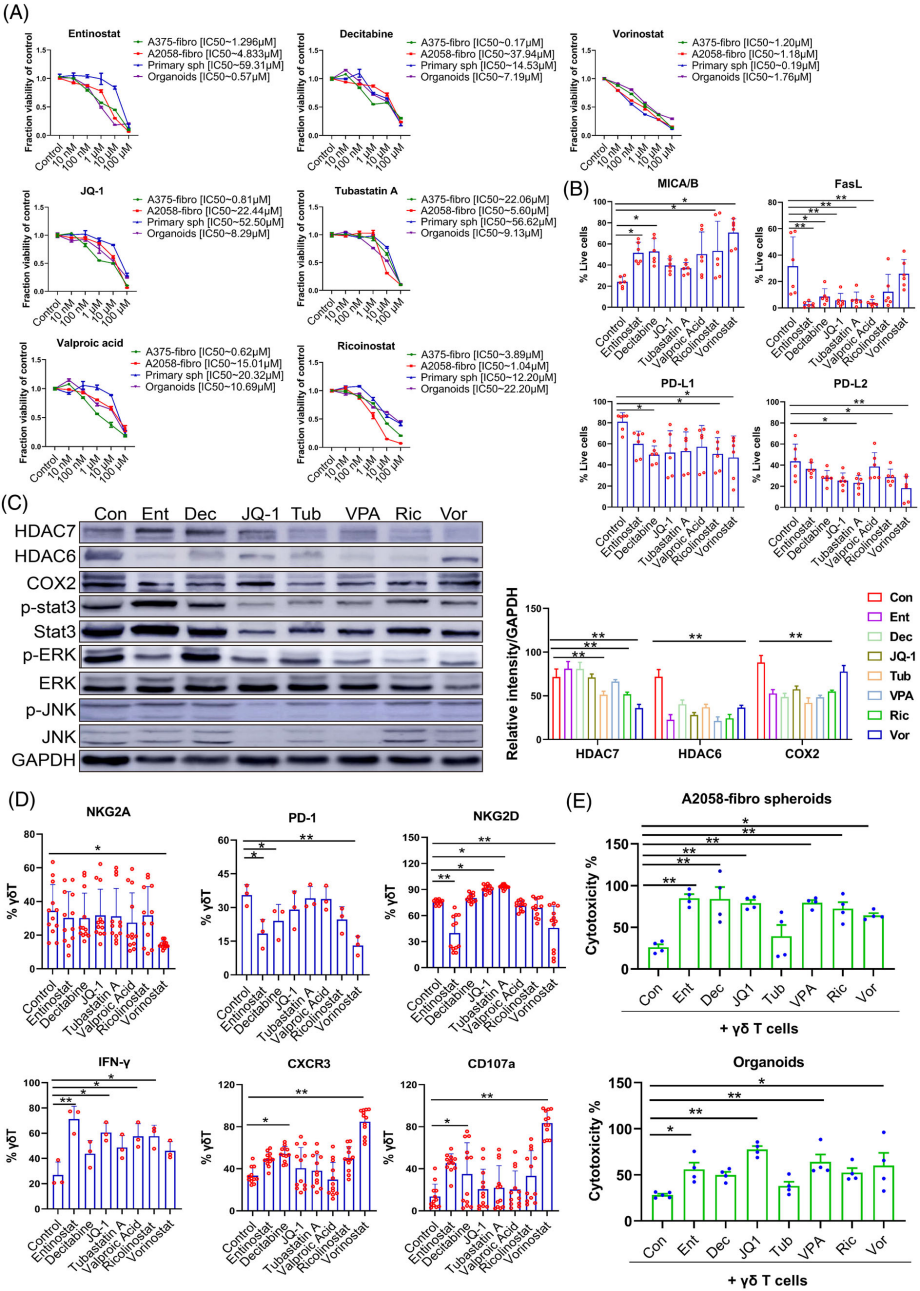
Epigenetic modifiers increase γδ T cell treatment efficacy. (A) Dose–response curves of bicellular A375‐fibroblast spheroids, A2058‐fibroblast spheroids, patient derived melanoma spheroids and melanoma patient‐derived organoids (MPDOs) after treatment with Entinostat, Decitabine, JQ‐1, Tubastatin A, Valproic acid, Ricolinostat, Vorinostat and DMSO controls for 3 days. Values represent mean ± SEM, *n* = 4. (B) Expression of MICA/B, FasL, PD‐L1 and PD‐L2 in cells from A2058‐fibroblast spheroids was measured by FACS after Entinostat, Decitabine, JQ‐1, Tubastatin A, Valproic acid, Ricolinostat and Vorinostat treatment (5 μM) for 48 h. *p < 0.05. **p < 0.01. (C) Western blot analysis of HDAC6, HDAC7, COX2, phospho‐STAT3, total STAT3, phosphor‐ERK, total ERK, phospho‐JNK, total JNK and GAPDH proteins in A2058‐fibroblast spheroids after Entinostat, Decitabine, JQ‐1, Tubastatin A, Valproic acid, Ricolinostat and Vorinostat treatment (5 μM) for 48 h (left panel). Densitometry quantification of the HDAC7, HDAC6 and COX2 (right panel). **p < 0.01. (D) NKG2A, PD‐1, NKG2D, CXCR3. CD107a and intracellular IFN‐γ in γδ T cells was measured by FACS after Entinostat, Decitabine, JQ‐1, Tubastatin A, Valproic acid, Ricolinostat and Vorinostat treatment (5 μM) for 48 h. *p < 0.05. **p < 0.01. (E) Cytotoxic function of γδ T cells against A2058‐fibro spheroids and MPDOs combined with 1 μM of Entinostat, Decitabine, JQ‐1, Tubastatin A, Valproic acid, Ricolinostat, Vorinostat and DMSO controls. *n* = 4. *p < 0.05. **p < 0.01

Many small molecules are being tested for the melanoma therapy. We aimed to discover small molecules that can be used to enhance the treatment efficacy of γδ T cells. We performed extensive literature search and identified 13 such potential compounds that across several classes, including epigenetic modifiers (Entinostat, Decitabine, JQ‐1, Tubastatin A, Valproic acid, Ricolinostat and Vorinostat), CDK inhibitor (Palbociclib and Seliciclib), CSF1R inhibitor GW2580, FGFR inhibitor Rogaratinib, and CXCR inhibitors (SB265610 and AMD3100). We first tested the effect of these compounds by themselves in melanoma–fibroblast bicellular spheroids, patient‐derived multicellular spheroids and MPDOs using CCK‐8 assay. These compounds inhibited the viability of cells in the 3D cultures at different concentrations. The IC50 value of the compounds in 3D cells were demonstrated in Figures 7A and S7A.

Epigenetic mediators are vital factors involved in maintaining an immunosuppressive TME that restrains antitumor immunity.^34^, ^35^ It has been shown that tumour cells and CAFs escape immune cell attack by expressing high levels of PD‐L1 and PD‐L2, and shedding NKG2D ligands (MICA, MICB and ULBP1‐6) from the cell surface through proteolytic cleavage.^36^ In addition, the expression of Fas ligand (FasL) on melanomas^37^ and CAFs^38^ has been implicated in their evasion of immune surveillance. We discovered that epigenetic modifiers, Entinostat, Decitabine, Ricolinostat and Vorinostat increased the expression of MICA/MICB on the cells in melanoma–fibroblast bicellular spheroids. Entinostat, Decitabine, JQ‐1, Tubastatin A and Valproic acid decreased FasL expression; Decitabine, Ricolinostat and Vorinostat decreased PD‐L1 expression, while Tubastatin A, Ricolinostat and Vorinostat decreased PD‐L2 expression in the spheroids (Figure 7B).

HDAC6, HDAC7 and STAT3/COX2 pathways are involved in tumour invasiveness and the immunosuppressive properties of CAFs.^35^, ^39^, ^40^ Western blot analysis of cells in the bicellular spheroids demonstrated that Tubastatin A, Ricolinostat and Vorinostat significantly decreased the expression of HDAC7; Entinostat, Decitabine, JQ‐1, Tubastatin A, Valproic acid and Ricolinostat significantly decreased the expression of HDAC6 and COX2; and several of the epigenetic modifiers inhibited p‐STAT3, as well as the downstream effector proteins, p‐ERK and p‐JNK (Figures 7C and Figure S7B).

We examined the effects of epigenetic modifiers on γδ T cells. Vorinostat significantly decreased the expression level of NKG2A; Entinostat, Decitabine and Vorinostat reduced the expression of PD‐1; JQ‐1 and Tubastatin A increased the expression of NKG2D; Entinostat, JQ‐1, Valproic acid and Ricolinostat increased the production of IFN‐γ; Decitabine and Vorinostat increased CXCR3 expression; and Decitabine and Vorinostat increased CD107a expression in γδ T cells (Figure 7D).

Next, the expanded γδ T cells were co‐cultured with melanoma–fibroblast bicellular spheroids and MPDOs, respectively. To study whether epigenetic modifiers enhance the cytotoxicity of γδ T cells, LDH release from spheroids and MPDOs was measured by the CytoTox 96® Non‐Radioactive Cytotoxicity Assay. We found that Entinostat, Decitabine, JQ‐1, Valproic acid, Ricolinostat and Vorinostat increased the cytotoxicity of γδ T cells against A2058‐fibroblast bicellular spheroids. Entinostat, Decitabine, JQ‐1, Valproic acid and Vorinostat significantly enhanced the cytotoxicity of γδ T cells against MPDOs (Figure 7E).

## 3 DISCUSSION

The treatment efficacy of γδ T cell immunotherapy is modest despite a satisfactory safety profile.^41^ Since Vδ2 T cells are unique to primates, a lack of adequate preclinical models hampers the development of Vδ2 T cell‐based therapy. Using the four different 3D cultured tumour models, we discovered that γδ T cells migrate to and infiltrate tumour spheroids more rapidly than CD8^+^ T cells. However, tumor infiltrating γδ T cells demonstrated exhausted phenotypes with limited tumour killing capacity. ICB enhanced tumour killing capacity of γδ T cells. In addition, we found that epigenetic modifiers, such as Entinostat and Vorinostat, increase γδ T cell functions through upregulation of MICA/MICB and downregulation of PD‐L1 in CAFs and melanoma cells; while downregulation of PD‐1, upregulation of CXCR3 and CD107a expression and IFN‐γ production in γδ T cells.

We find that both αβ and γδ T cells in the melanoma spheroids express high level of checkpoint proteins. The addition of fibroblasts in the melanoma spheroids makes the spheroids more immunosuppressive and significantly reduces spheroid infiltrating immune cells. MPDOs retain the complex diversity of melanoma, and preserve their native infiltrating immune cell populations, including lymphocytes and myeloid cells. The immunosuppressive TME of these 3D models is resulted not only from tumour cells but also from CAFs, lymphocytes and myeloid cells, accompanied by high expression of PD‐L1 and PD‐L2 with reduced expression of MICA/B, similar to those in patient melanoma tissues.^42^, ^43^ From unicellular tumor spheroids to MPDOs, these 3D models escalate complexity of tum or stroma, which may provide preclinical platforms to meet different measurement needs for immunotherapy studies.

γδ T cells serve as sentinels in the innate immune system and play a critical role in cancer immunosurveillance.^44^, ^45^ We find that γδ T cells can migrate to and infiltrate melanoma spheroids more quickly and better than CD8^+^ T cells. γδ TCR can directly recognise general stress ligands, such as phosphoantigens in the tumour cells, without priming. While activation of αβ T cells generally requires antigen presenting cells and priming. The advantage of γδ T cells over αβ T cells in infiltrating tumour spheroids is in line with other studies showing that the tumour‐infiltrating γδ T cells product an early source of IFN‐γ that in turn may regulate the function of tumour‐triggered αβ T cells.^46^ γδ T cells are endowed with a variety of effector functions, and the γδ T cells infiltrated in spheroids possess effector memory phenotype that is a signature of adaptive immune response and known capable of killing melanoma cells.

Even though γδ T cells can infiltrate tumor spheroids, they quickly show exhausted immunophenotypes by increased expression of CTLA‐4, PD‐1 and PD‐L1 with limited tumor killing capacity in the spheroids. This phenomenon may explain why adoptive transfer of γδ T cells had limited efficacy in many clinical trials. This is expected perhaps as the immune suppressive TME in patients inhibits γδ T cell functions. To overcome the inhibition, we test the combination of γδ T cells with PD‐1 and/or CTLA‐4 mAbs in all the models. We discover that anti‐PD1 mAbs and the combination with anti‐CTLA‐4/PD‐1 mAbs significantly enhance γδ T cell functions, decrease size of spheroids and increase tumour apoptosis. Our data support that ICB improves treatment efficacy of γδ T cells.

Using the 3D models, we also test small molecules that may enhance the treatment efficacy of γδ T cells. We find that epigenetic modifiers can significantly improve γδ T cell anti‐tumour function in spheroid models. Tubastatin‐A, Ricolinostat and Vorinostat significantly inhibit HDAC6/7 pathways that are involved in tumour proliferation and migration in CAFs and melanoma cells. In advanced cancers, tumour cells shed NKG2D ligand for immune evasion.^47^ We discover that Entinostat,^48^, ^49^ Decitabine,^50^, ^51^ Ricolinostat and Vorinostat significantly increase MICA and MICB expression on tumour cells and CAFs. Vorinostat significantly decrease NKG2A expression, while JQ‐1 and Tubastatin‐A increase NKG2D expression in γδ T cells. Therefore, these epigenetic modifiers may inhibit melanoma immune evasion. In addition, our results demonstrated that several of these epigenetic modifiers increased γδ T cell function by increasing IFN‐γ production, CXCR3 and CD107a expression, and decreasing the expression of PD‐1 in γδ T cells.

In conclusion, spheroid/MPDO models are useful preclinical platforms for studying interaction of tumour cells, stromal cells and γδ T cells. ICB enhances the therapeutic efficacy of γδ T cells by inhibiting their exhausted immunophenotypes. Epigenetic modifiers increase γδ T cells’ anti‐tumour function by inhibiting tumour proliferation and immune evasion as well as enhancing γδ T chemotaxis and cytokine production.

## 4 MATERIAL AND METHODS

### 4.1 Cell lines

The melanoma cell lines A2058 cells, UACC903 cells and A375 cells were gifted from Meenhard Herlyn’ lab at the Wistar Institute. Cells were cultured in 1640 RPMI medium (Gibco, Grand Island, NY) contained with 10% FBS (Gibco) and 1% penicillin/streptomycin (Gibco). The human fibroblasts cell line GM01948 and AG11732A (gifted from the Wistar Institute) were maintained in DMEM medium (Gibco). All cells were cultured at 37°C and 5% CO_2_.

### 4.2 Isolation of peripheral blood mononuclear cells, γδ T cell expansion and CD8 T cell activation

PBMCs and pure CD8 T cells were isolated from peripheral blood of healthy donors provided by Immunology Core at the University of Pennsylvania with informed consent and ethical approval (Protocol number 707906). For γδ T expansion, PBMCs were seeded at in 24‐well plate (2 × 10^6^ cells/well) and cultured with RPMI 1640 medium contained with IL‐2 (200 IU/ml, PeproTech, NJ) and Zoledronic acid (5 μM, Sigma‐Aldrich, USA).^52^ And for CD8 T cells activation, pure CD8 T cells were stimulated by anti‐CD3 (2 μg/ml, Biolegend, USA) and anti‐CD28 (2 μg/ml, Biolegend) for 12 h as previously described.^29^ The expanded γδ T cells and CD8 T cells were collected from the same donors and compared in the experiments.

### 4.3 Spheroid generation and co‐culture

To generate unicellular spheroids, 50 μl 1.5% agarose gel (A9539, Sigma‐Aldrich, St. Louis, Missouri) were pre‐coated on 96‐well plate and melanoma cells (2 × 10^4^) were seeded on agarose immediately centrifuged at 180 × g for 3 min. To generate tumour–fibroblast bicellular spheroids, tumour cells (A2058 cells, UACC903 cells and A375 cells) and fibroblasts were mixed at a 1:2 ratio and seeded on agarose. Spheroid formation was observed after overnight incubation. After 48 h incubation, spheroids were co‐cultured with PBMCs, expanded γδ T cells or activated CD8 T cells (tumour cell: T cell ratio is 1:2). In some experiments, 50 μg/ml isotype control IgG, 50 μg/ml anti‐PD‐1 (Pembrolizumab) or 50 μg/ml anti‐CTLA‐4 (Ipilimumab) were used.

### 4.4 Generation of patient‐derived multicellular spheroids

Fresh tumour specimens were collected under the Abramson Cancer Center's melanoma research program tissue collection protocol UPCC 08607 in accordance with the Institutional Review Board of the University of Pennsylvania. De‐identified tumours were harvested from patients with stage III– IV melanoma. Both male and female melanoma patients between the ages of 26–86 years old were included in this study. Melanoma tissues were minced on ice and then digested in complete RPMI supplemented with 200 U/ml collagenase IV (Sigma) and 50 U/ml DNase I (Sigma) for 30 min at 37°C. The supernatant was then filtered through a 100 μm strainers (Falcon, BD Biosciences) and centrifuged at 400 × *g*. The cell pellet was washed by L15 medium (containing 10% FBS, 1% penicillin/streptomycin) for three times to ensure removal of the proteases. The culture medium is followed: 400 ml MCDB‐153 medium; 100 ml L15 medium; 10 ml FBS; 560 μl CaCl2 (initial concentration of 1.68 mM), 0.5 ml insulin (2.5 μg/ml); 0.5 ml EGF (Sigma, E‐4127, 5 μg/ml); 1 ml BPE (Gibco, 13028014). After culture for 14 days, the adherent cells were trypsinised and seeded on agarose in 96‐well plates to create a unique uniform spheroid. The spheroids were co‐cultured with PBMCs or expanded γδ T cells and subsequent experiments were done as tumour cell lines.

### 4.5 Generation of melanoma patient‐derived organoids

Fresh melanoma samples were washed with cold PBS (supplement with 2% penicillin/streptomycin) and then minced finely. Fragments were digested with 200 U/ml collagenase IV and 50 U/ml DNase I for 30 min at 37°C. The digested cell mixture was diluted in medium and filtered by the 100 μm strainers (Falcon). And the single‐cell suspension was seeded on the solidified Matrigel in 24‐well plates and cultured for up to 7–10 days for subsequent analyses. The organoids were cultured with ADMEM/F12 medium contained of 1% P/S, 50% conditioned media of L‐WRN cell line (ATCC), 0.5 mM A83‐01 (Tocris), 10 mM Nicotinamide (Sigma), 1 mM *N*‐Acetylcysteine (Sigma), B‐27 without vitamin A (Invitrogen), 50 ng/ml EGF (Invitrogen), 50 ng/ml FGF (Peprotech) and 10 ng/ml Forskolin (Tocris).

### 4.6 Small molecule screening

After extensive literature search, 13 compounds were selected to test their effects on tumour spheroids and MPDOs, including Entinostat, Decitabine, Ricolinostat, Vorinostat, Valproic acid, JQ‐1, Tubastatin‐A, Rogaratinib, Palbociclib, Seliciclib, GW2580, SB265610, AMD3100 and dimethyl sulfoxide (DMSO) control. These compounds were provided by Dr. Meenhard Herlyn's lab at the Wistar Institute. The tumour–fibroblast spheroids and organoids were seeded onto 96‐well white plates (5 × 10^3^ cells per a well) to grow for 4 days, the compounds were diluted in a range of concentrations and dispensed in the culture medium, and cell viability was assayed using CCK‐8 kit after 3 days of drug incubation without γδ T cells. The cell viability measurement was conducted in triplicate and repeated independently one time. The cytotoxicity assays of γδ T cells and compounds combination against spheroids and organoids were tested by the CytoTox 96® Non‐Radioactive Cytotoxicity Assay, the working concentration of compounds were 1 μM. The activity of lactate dehydrogenase (LDH) released by spheroids and organoids was measured. The LDH measurement was conducted in four replicates and repeated independently one time.

(1) $$\%\;\mathrm{cytotoxicity}\;=\;100\;\times \frac{\mathrm{Experimental}-\mathrm{effector}\;\mathrm{spontaneous}-\mathrm{target}\;\mathrm{spontaneous}\;}{\mathrm{Target}\;\mathrm{maximum}-\mathrm{target}\;\mathrm{spontaneous}}.$$

### 4.7 Antibodies and flow cytometry analyses

For FACS analyses, six spheroids were collected and sediment to the bottom of the 1.5 ml tubes. Subsequently, the non‐infiltrating immune cells (defined as outside of spheroids) in the supernatant cell suspension was collected. Spheroids were then dissected by Trypsin (Gibco) to obtain a single‐cell suspension (the immune cells defined as inside of spheroids). The outside and inside compartments were further measured by FACS. For the cell counting of PBMCs or γδ T cells, cells were prelabelled with CFSE before co‐cultured with spheroids, spheroids were gently resuspended and dissected by Trypsin (Gibco) to obtain a single‐cell suspension for cell counting by flow cytometry. The fluorescent‐labelled antibodies used for flow cytometry analyses including anti‐CD45 (2D1), anti‐CD3 (UCHT1), anti‐CD4 (OKT4), anti‐CD8 (SK1), anti‐CD45RA (HI100), anti‐CD27 (LG.3A10), anti‐NKG2D (1D11), anti‐IFN‐γ (B27), anti‐PD‐1 (EH12.1), anti‐CTLA‐4 (BNI3), anti‐PD‐L1(10F.9G2), anti‐TCRγ/δ (B1), anti‐Vδ2 (B6), anti‐CD14 (63D3), anti‐CD11b (ICRF44), anti‐CD277 (232‐5, Biosciences), anti‐CD69 (FN50), anti‐TGF‐β1 (TW4‐2F8), anti‐MICA/B (6D4), anti‐FasL (NOK‐1), anti‐PD‐L1 (29E.2A3), anti‐PD‐L2 (MIH18), anti‐NKG2A (S19004C), anti‐CXCR3 (G025H7) and anti‐CD107a (H4A3), all from BD Biosciences or Biolegend. And the fluorochromes and catalogue number were listed in Table S1. The staining cells were measured by BD flow cytometer (BD Biosciences, Baltimore, MD) and the results were analysed by FlowJo software (Tree Star, OR, USA).

### 4.8 Immunofluorescence and spheroid volume calculation

Before starting the co‐cultures, PBMCs or expanded γδ T cells were stained with CFSE (Thermo Fisher Scientific, San Jose, CA), tumour cells were stained with Cell Tracker™ Orange (Thermo Fisher Scientific) and fibroblasts were stained with Cell Tracker™ violet (Thermo Fisher Scientific) following working procedure. Infiltrated spheroids were isolated and fixed with 4% paraformaldehyde overnight. All images were taken on a Zeiss LSM 710 (Carl Zeiss, Thornwood, New York). Images were then analysed by measuring the major axial length and the minor axial length (taken as width) of each spheroid. The volumes of spheroid were calculated as follows: *V* = 0.5 × length × width^2^.^53^, ^54^ And the antibodies were used for IF including anti‐HMB45 (ab733, abcam), Alexa Fluor™ 488 Phalloidin (A12379, Invitrogen), ICAM‐1 (10020‐1‐AP, Proteintech), Alpha‐Smooth Muscle Actin Monoclonal Antibody (α‐SMA, 14‐9760‐80, eBioscience), FAP Antibody (AF3715‐SP, R&D Systems), Vimentin (5741T, Cell Signaling Technology) and CD45 (13917s, Cell Signaling Technology).

### 4.9 Live/dead staining assay

The ViaStain AOPI Staining (Nexcelom, CS2‐0106) were used for live/dead staining after γδ T cells co‐cultured with organoids for 48 h. The organoids were incubated with AOPI dyes for 20 min at 37°C in the dark. The images were taken by Zeiss LSM 710 (Carl Zeiss) and analysed by the ZEN software.

### 4.10 Apoptosis assay

Apoptotic cells were measured after spheroids or organoids co‐culture with γδ T cells for 48 h. Spheroids and organoids were dissected by Trypsin (Gibco). The single‐cell suspension was washed with cold PBS for twice and resuspended in 100 μl binding buffer contained of 5 μl of AV‐FITC and 7‐AAD solution (Biolegend). The cells were incubated for 15 min in the dark on ice. FACS was performed to measure the staining of AV and 7‐AAD.

### 4.11 Western blot analysis

Cells in spheroids were lysed and the whole cell lysates were determined by10% sodium dodecyl sulphate polyacrylamide gel electrophoresis and polyvinylidene difluoride (PVDF) membranes transfer. The membranes were incubated with specific primary antibodies at 4°C overnight and HRP‐conjugated secondary antibodies at room temperature for 2 h. The primary antibodies were including rabbit anti‐HDAC6 (#7558), HDAC7 (#10831), total STAT3 (#12640), total ERK (#4695), total JNK (#9258), phospho‐STAT3 (#9145), phosphor‐ERK (#4370), phospho‐JNK (#4668), COX2 (#12282) and GAPDH (Cell Signalling Technology, Beverly, MA, USA).

### 4.12 Statistical analysis

Data are analysed by Prism 9.0 of GraphPad Software. The Student's *t*‐test was used to analyse the differences between two groups, and the one‐way ANOVA with multiple comparisons were used to assess the differences in several groups. *P*‐values < 0.05 were considered significant difference.

## Supporting information

Supporting Information

Supporting Information

Supporting Information

Supporting Information

Supporting Information

Supporting Information

Supporting Information

## References

[1] Van Acker HH , Anguille S , Van Tendeloo VF , Lion E . Empowering gamma delta T cells with antitumor immunity by dendritic cell‐based immunotherapy. Oncoimmunology. 2015;4(8):e1021538. 26405575 10.1080/2162402X.2015.1021538 PMC4570126

[2] Girard P , Charles J , Cluzel C , et al. The features of circulating and tumor‐infiltrating gammadelta T cells in melanoma patients display critical perturbations with prognostic impact on clinical outcome. Oncoimmunology. 2019;8(8):1601483. 31413911 10.1080/2162402X.2019.1601483 PMC6682366

[3] Van Acker HH , Anguille S , Willemen Y , Van den Bergh JM , Berneman ZN , Lion E , et al. Interleukin‐15 enhances the proliferation, stimulatory phenotype, and antitumor effector functions of human gamma delta T cells. J Hematol Oncol. 2016;9(1):101. 27686372 10.1186/s13045-016-0329-3 PMC5041439

[4] Deniger DC , Moyes JS , Cooper LJ . Clinical applications of gamma delta T cells with multivalent immunity. Front Immunol. 2014;5:636. 25566249 10.3389/fimmu.2014.00636 PMC4263175

[5] Pitt JM , Vetizou M , Daillere R , et al. Resistance mechanisms to immune‐checkpoint blockade in cancer: Tumor‐intrinsic and ‐extrinsic factors. Immunity. 2016;44(6):1255‐1269. 27332730 10.1016/j.immuni.2016.06.001

[6] Sullivan LC , Shaw EM . Westall GP. gammadelta T cells in transplantation: Friend and foe. Transplantation. 2018;102(12):1970‐1971. 29994979 10.1097/TP.0000000000002336

[7] Varesano S , Zocchi MR , Poggi A . Zoledronate triggers vdelta2 T cells to destroy and kill spheroids of colon carcinoma: Quantitative image analysis of three‐dimensional cultures. Front Immunol. 2018;9:998. 29867975 10.3389/fimmu.2018.00998 PMC5951939

[8] Grivennikov SI , Greten FR , Karin M . Immunity, inflammation, and cancer. Cell. 2010;140(6):883‐899. 20303878 10.1016/j.cell.2010.01.025 PMC2866629

[9] Erdogan B , DJ Webb . Cancer‐associated fibroblasts modulate growth factor signaling and extracellular matrix remodeling to regulate tumor metastasis. Biochem Soc Trans. 2017;45(1):229‐236. 28202677 10.1042/BST20160387 PMC5371349

[10] Gascard P , Tlsty TD . Carcinoma‐associated fibroblasts: Orchestrating the composition of malignancy. Genes Dev. 2016;30(9):1002‐1019. 27151975 10.1101/gad.279737.116 PMC4863733

[11] Costa A , Kieffer Y , Scholer‐Dahirel A , et al. Fibroblast heterogeneity and immunosuppressive environment in human breast cancer. Cancer Cell. 2018;33(3):463‐479. 29455927 10.1016/j.ccell.2018.01.011

[12] Fiori ME , Di Franco S , Villanova L , Bianca P , Stassi G , De Maria R . Cancer‐associated fibroblasts as abettors of tumor progression at the crossroads of EMT and therapy resistance. Mol Cancer. 2019;18(1):70. 30927908 10.1186/s12943-019-0994-2 PMC6441236

[13] Pauza CD , Liou ML , Lahusen T , et al. Gamma delta T cell therapy for cancer: It is good to be local. Front Immunol. 2018;9:1305. 29937769 10.3389/fimmu.2018.01305 PMC6003257

[14] Zhao X , Subramanian S . Intrinsic resistance of solid tumors to immune checkpoint blockade therapy. Cancer Res. 2017;77(4):817‐822. 28159861 10.1158/0008-5472.CAN-16-2379

[15] Clevers H . Modeling development and disease with organoids. Cell. 2016;165(7):1586‐1597. 27315476 10.1016/j.cell.2016.05.082

[16] Hirschhaeuser F , Menne H , Dittfeld C , West J , Mueller‐Klieser W , Kunz‐Schughart LA . Multicellular tumor spheroids: An underestimated tool is catching up again. J Biotechnol. 2010;148(1):3‐15. 20097238 10.1016/j.jbiotec.2010.01.012

[17] Kunz‐Schughart LA , Freyer JP , Hofstaedter F , Ebner R . The use of 3‐D cultures for high‐throughput screening: The multicellular spheroid model. J Biomol Screen. 2004;9(4):273‐285. 15191644 10.1177/1087057104265040

[18] Jenkins RW , Aref AR , Lizotte PH , et al. Ex vivo profiling of PD‐1 blockade using organotypic tumor spheroids. Cancer Discov. 2018;8(2):196‐215. 29101162 10.1158/2159-8290.CD-17-0833 PMC5809290

[19] Lanuza PM , Vigueras A , Olivan S , et al. Activated human primary NK cells efficiently kill colorectal cancer cells in 3D spheroid cultures irrespectively of the level of PD‐L1 expression. Oncoimmunology. 2018;7(4):e1395123. 29632716 10.1080/2162402X.2017.1395123 PMC5889279

[20] Hirt C , Papadimitropoulos A , Mele V , et al. In vitro 3D models of tumor‐immune system interaction. Adv Drug Deliv Rev. 2014;79‐80:145‐154. 10.1016/j.addr.2014.05.003 24819215

[21] Musso A , Catellani S , Canevali P , et al. Aminobisphosphonates prevent the inhibitory effects exerted by lymph node stromal cells on anti‐tumor Vdelta 2 T lymphocytes in non‐Hodgkin lymphomas. Haematologica. 2014;99(1):131‐139. 24162786 10.3324/haematol.2013.097311 PMC4007944

[22] Zocchi MR , Costa D , Vene R , et al. Zoledronate can induce colorectal cancer microenvironment expressing BTN3A1 to stimulate effector gammadelta T cells with antitumor activity. Oncoimmunology. 2017;6(3):e1278099. 28405500 10.1080/2162402X.2016.1278099 PMC5384426

[23] Brás MM , Radmacher M , Sousa SR , Granja PL . Melanoma in the eyes of mechanobiology. Front Cell Dev Biol. 2020;8:54. 32117980 10.3389/fcell.2020.00054 PMC7027391

[24] Liu T , Zhou L , Yang K , et al. The β‐catenin/YAP signaling axis is a key regulator of melanoma‐associated fibroblasts. Signal Transduct Target Ther. 2019;4:63. 31885878 10.1038/s41392-019-0100-7 PMC6928146

[25] Budhu S , Loike JD , Pandolfi A , et al. CD8+ T cell concentration determines their efficiency in killing cognate antigen‐expressing syngeneic mammalian cells in vitro and in mouse tissues. J Exp Med. 2010;207(1):223‐235. 20065066 10.1084/jem.20091279 PMC2812553

[26] Giannattasio A , Weil S , Kloess S , et al. Cytotoxicity and infiltration of human NK cells in in vivo‐like tumor spheroids. BMC Cancer. 2015;15:351. 25933805 10.1186/s12885-015-1321-y PMC4422268

[27] Jiang X , Xu J , Liu M , et al. Adoptive CD8(+) T cell therapy against cancer: Challenges and opportunities. Cancer Lett. 2019;462:23‐32. 31356845 10.1016/j.canlet.2019.07.017

[28] Kabelitz D , Serrano R , Kouakanou L , Peters C , Kalyan S . Cancer immunotherapy with γδ T cells: Many paths ahead of us. Cell Mol Immunol. 2020;17(9):925‐939. 32699351 10.1038/s41423-020-0504-x PMC7609273

[29] Guillerey C , Harjunpää H , Carrié N , et al. TIGIT immune checkpoint blockade restores CD8(+) T‐cell immunity against multiple myeloma. Blood. 2018;132(16):1689‐1694. 29986909 10.1182/blood-2018-01-825265

[30] Xu X , Davelaar N , Mus AM , et al. Interleukin‐17A is produced by CD4+ but not CD8+ T cells in synovial fluid following T cell receptor activation and regulates different inflammatory mediators compared to tumor necrosis factor in a model of psoriatic arthritis synovitis. Arthritis Rheumatol. 2020;72(8):1303‐1313. 32243724 10.1002/art.41271 PMC7497075

[31] Kobayashi H , Tanaka Y , Yagi J , Minato N , Tanabe K . Phase I/II study of adoptive transfer of gamma delta T cells in combination with zoledronic acid and IL‐2 to patients with advanced renal cell carcinoma. Cancer Immunol Immunother. 2011;60(8):1075‐1084. 21519826 10.1007/s00262-011-1021-7 PMC11029699

[32] Schultze JL , Anderson KC , Gilleece MH , Gribben JG , Nadler LM . A pilot study of combined immunotherapy with autologous adoptive tumour‐specific T‐cell transfer, vaccination with CD40‐activated malignant B cells and interleukin 2. Br J Haematol. 2001;113(2):455‐460. 11380416 10.1046/j.1365-2141.2001.02760.x

[33] Ou L , Wang H , Liu Q , et al. Dichotomous and stable gamma delta T‐cell number and function in healthy individuals. J Immunother Cancer. 2021;9(5):e002274. 34011536 10.1136/jitc-2020-002274 PMC8137237

[34] Kim DJ , Dunleavey JM , Xiao L , et al. Suppression of TGFβ‐mediated conversion of endothelial cells and fibroblasts into cancer associated (myo)fibroblasts via HDAC inhibition. Br J Cancer. 2018;118(10):1359‐1368. 29695769 10.1038/s41416-018-0072-3 PMC5959903

[35] Li A , Chen P , Leng Y , Kang J . Histone deacetylase 6 regulates the immunosuppressive properties of cancer‐associated fibroblasts in breast cancer through the STAT3‐COX_2_‐dependent pathway. Oncogene. 2018;37(45):5952‐5966. 29980788 10.1038/s41388-018-0379-9

[36] Chitadze G , Lettau M , Bhat J , et al. Shedding of endogenous MHC class I‐related chain molecules A and B from different human tumor entities: Heterogeneous involvement of the “a disintegrin and metalloproteases” 10 and 17. Int J Cancer. 2013;133(7):1557‐1566. 23526433 10.1002/ijc.28174

[37] Zhu J , Petit PF , Van den Eynde BJ . Apoptosis of tumor‐infiltrating T lymphocytes: A new immune checkpoint mechanism. Cancer Immunol Immunother. 2019;68(5):835‐847. 30406374 10.1007/s00262-018-2269-y PMC11028327

[38] Lakins MA , Ghorani E , Munir H , Martins CP , Shields JD . Cancer‐associated fibroblasts induce antigen‐specific deletion of CD8 (+) T cells to protect tumour cells. Nat Commun. 2018;9(1):948. 29507342 10.1038/s41467-018-03347-0 PMC5838096

[39] Jones DL , Haak AJ , Caporarello N , et al. TGFβ‐induced fibroblast activation requires persistent and targeted HDAC‐mediated gene repression. J Cell Sci. 2019;132(20):jcs233486. 31527052 10.1242/jcs.233486 PMC6826010

[40] Banik D , Noonepalle S , Hadley M , et al. HDAC6 plays a noncanonical role in the regulation of antitumor immune responses, dissemination, and invasiveness of breast cancer. Cancer Res. 2020;80(17):3649‐3662. 32605998 10.1158/0008-5472.CAN-19-3738 PMC7484424

[41] Zhao Y , Niu C , Cui J . Gamma‐delta (gammadelta) T cells: Friend or foe in cancer development?. J Transl Med. 2018;16(1):3. 29316940 10.1186/s12967-017-1378-2 PMC5761189

[42] Oatmen KE , Cull E , Spinale FG . Heart failure as interstitial cancer: Emergence of a malignant fibroblast phenotype. Nat Rev Cardiol. 2020;17(8):523‐531. 31686012 10.1038/s41569-019-0286-y

[43] Lynch MD , Watt FM . Fibroblast heterogeneity: Implications for human disease. J Clin Invest. 2018;128(1):26‐35. 29293096 10.1172/JCI93555 PMC5749540

[44] Dieli F , Stassi G , Todaro M , Meraviglia S , Caccamo N , Cordova A . Distribution, function and predictive value of tumor‐infiltrating γδ T lymphocytes. Oncoimmunology. 2013;2(4):e23434. 23734305 10.4161/onci.23434 PMC3654575

[45] Ma C , Zhang Q , Ye J , et al. Tumor‐infiltrating γδ T lymphocytes predict clinical outcome in human breast cancer. J Immunol. 1950;189(10):5029‐5036. 10.4049/jimmunol.1201892 PMC4832413 23034170

[46] Gao Y , Yang W , Pan M , et al. Gamma delta T cells provide an early source of interferon gamma in tumor immunity. J Exp Med. 2003;198(3):433‐442. 12900519 10.1084/jem.20030584 PMC2194096

[47] Paczulla AM , Rothfelder K , Raffel S , et al. Absence of NKG2D ligands defines leukaemia stem cells and mediates their immune evasion. Nature. 2019;572(7768):254‐259. 31316209 10.1038/s41586-019-1410-1 PMC6934414

[48] Hicks KC , Knudson KM , Lee KL , et al. Cooperative immune‐mediated mechanisms of the HDAC inhibitor entinostat, an IL15 superagonist, and a cancer vaccine effectively synergize as a novel cancer therapy. Clin Cancer Res. 2020;26(3):704‐716. 31645354 10.1158/1078-0432.CCR-19-0727 PMC8274944

[49] Pili R , Quinn DI , Hammers HJ , et al. Immunomodulation by entinostat in renal cell carcinoma patients receiving high‐dose interleukin 2: A multicenter, single‐arm, phase I/II trial (NCI‐CTEP#7870). Clin Cancer Res. 2017;23(23):7199‐7208. 28939740 10.1158/1078-0432.CCR-17-1178 PMC5712266

[50] Wang Z , Wang Z , Li S , et al. Decitabine enhances Vγ9Vδ2 T cell‐mediated cytotoxic effects on osteosarcoma cells via the NKG2DL–NKG2D axis. Front Immunol. 2018;9:1239. 29910819 10.3389/fimmu.2018.01239 PMC5992281

[51] Reid MA , Dai Z , Locasale JW . The impact of cellular metabolism on chromatin dynamics and epigenetics. Nat Cell Biol. 2017;19(11):1298‐1306. 29058720 10.1038/ncb3629 PMC5886854

[52] Castella B , Kopecka J , Sciancalepore P , et al. The ATP‐binding cassette transporter A1 regulates phosphoantigen release and Vgamma9Vdelta2 T cell activation by dendritic cells. Nat Commun. 2017;8:15663. 28580927 10.1038/ncomms15663 PMC5465356

[53] Ayers GD , McKinley ET , Zhao P , et al. Volume of preclinical xenograft tumors is more accurately assessed by ultrasound imaging than manual caliper measurements. J Ultrasound Med. 2010;29(6):891‐901. 20498463 10.7863/jum.2010.29.6.891 PMC2925269

[54] Chen W , Wong C , Vosburgh E , Levine AJ , Foran DJ , Xu EY . High‐throughput image analysis of tumor spheroids: A user‐friendly software application to measure the size of spheroids automatically and accurately. J Vis Exp. 2014;89:51639. 10.3791/51639 PMC4212916 25046278

