# Supplementary figures and images for: Preclinical platforms to study therapeutic efficacy of human γδ T cells
Source: Clin Transl Med. 2022 Jun 22;12(6):e814. doi: 10.1002/ctm2.814 (PMC9217106; doi:10.1002/ctm2.814)

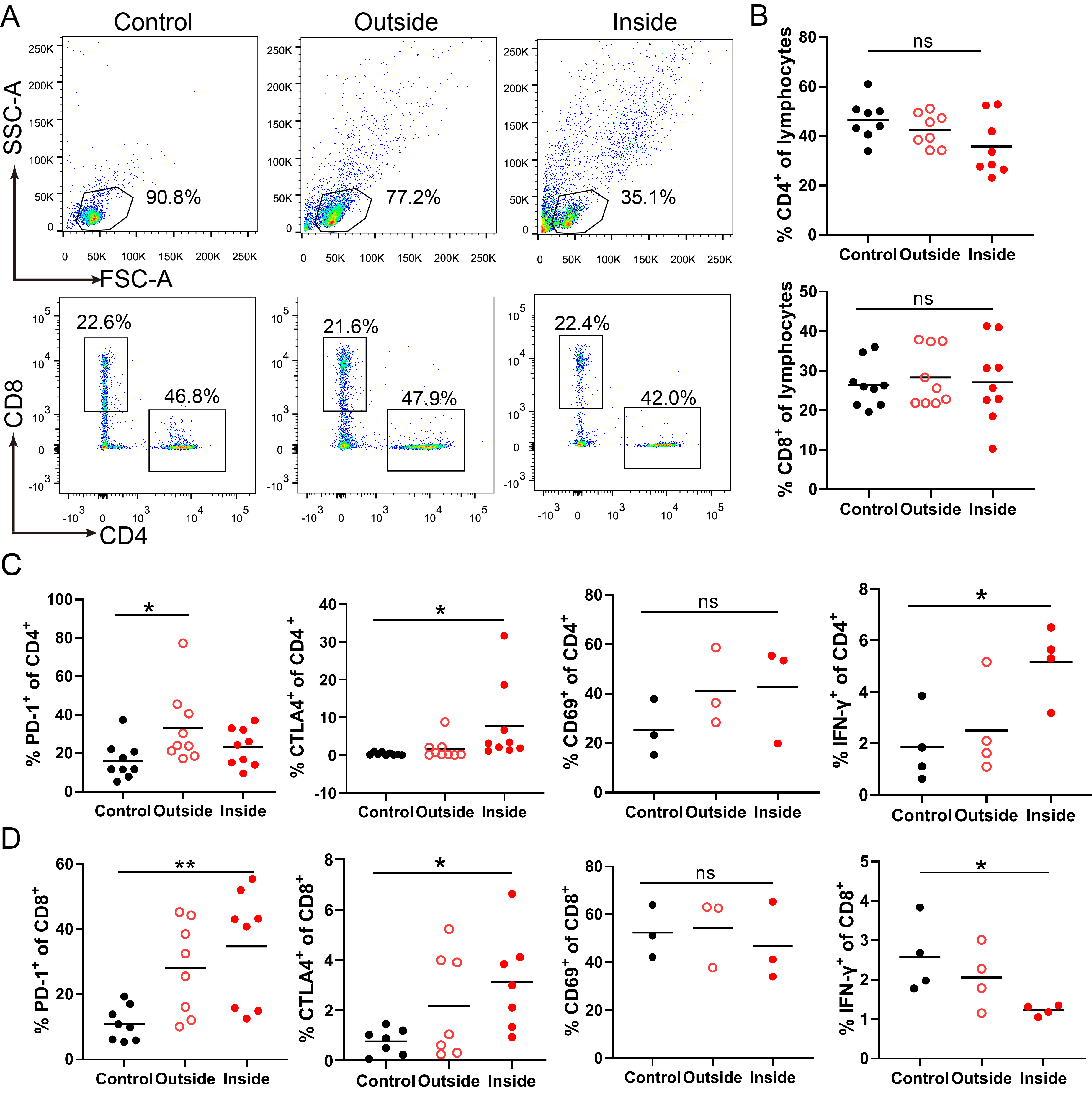

Supplement: Supplementary file 1 — Supporting Information [file CTM2-12-e814-s007.jpg]

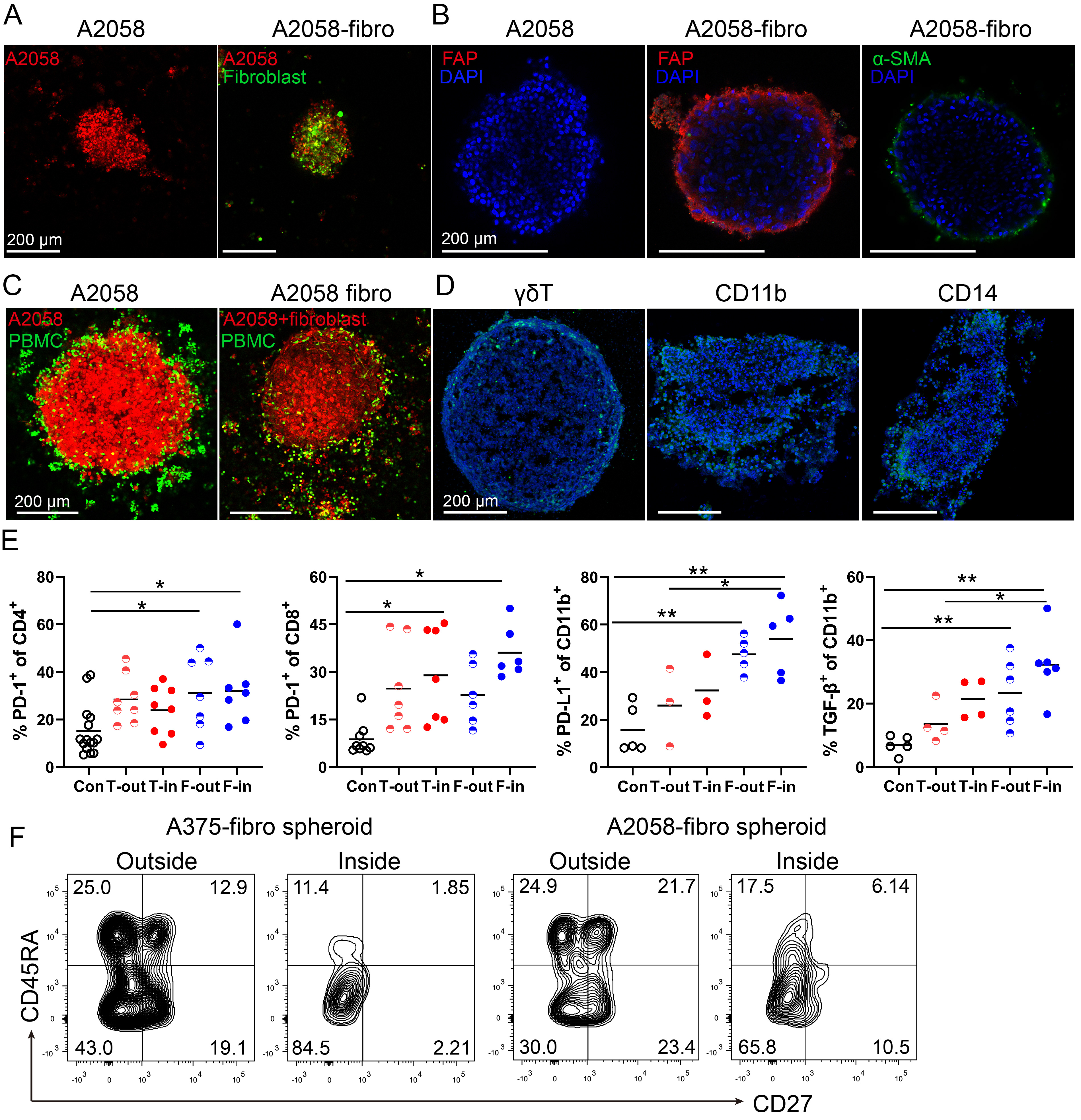

Supplement: Supplementary file 2 — Supporting Information [file CTM2-12-e814-s001.jpg]

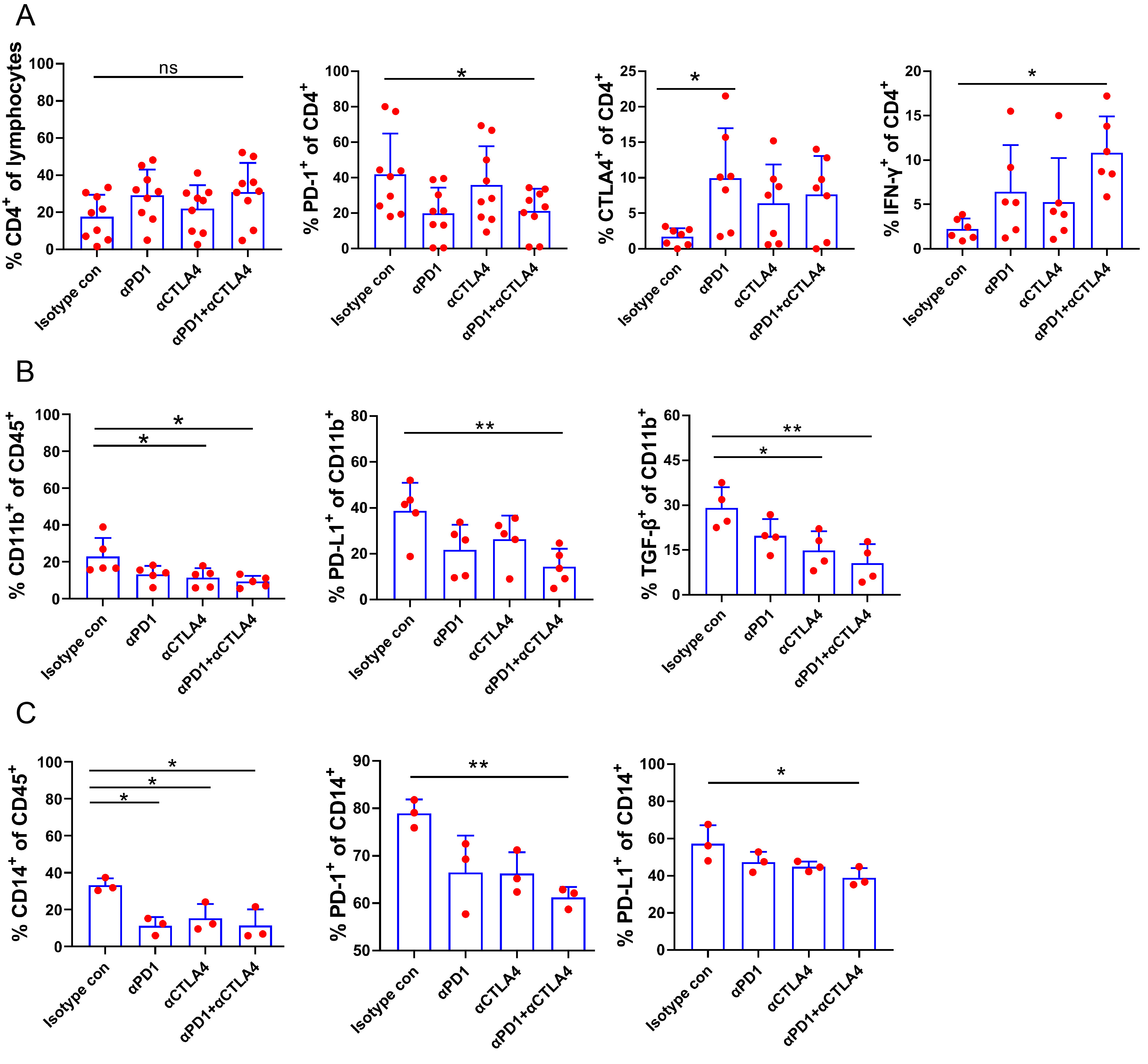

Supplement: Supplementary file 3 — Supporting Information [file CTM2-12-e814-s004.jpg]

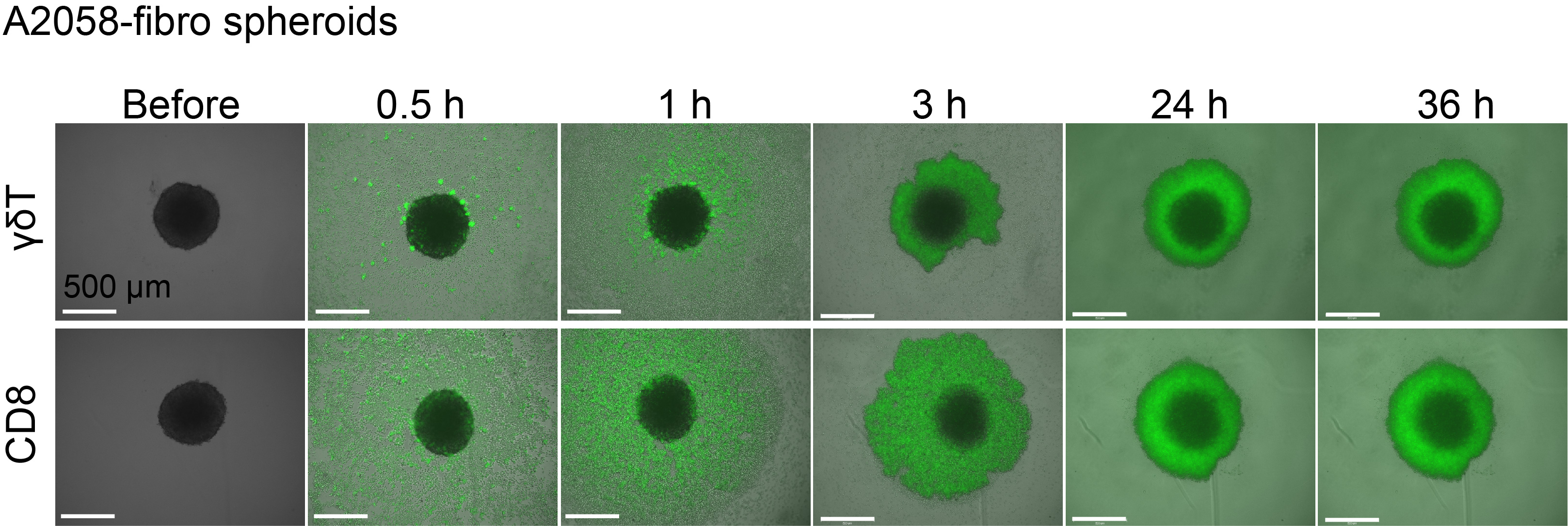

Supplement: Supplementary file 4 — Supporting Information [file CTM2-12-e814-s002.jpg]

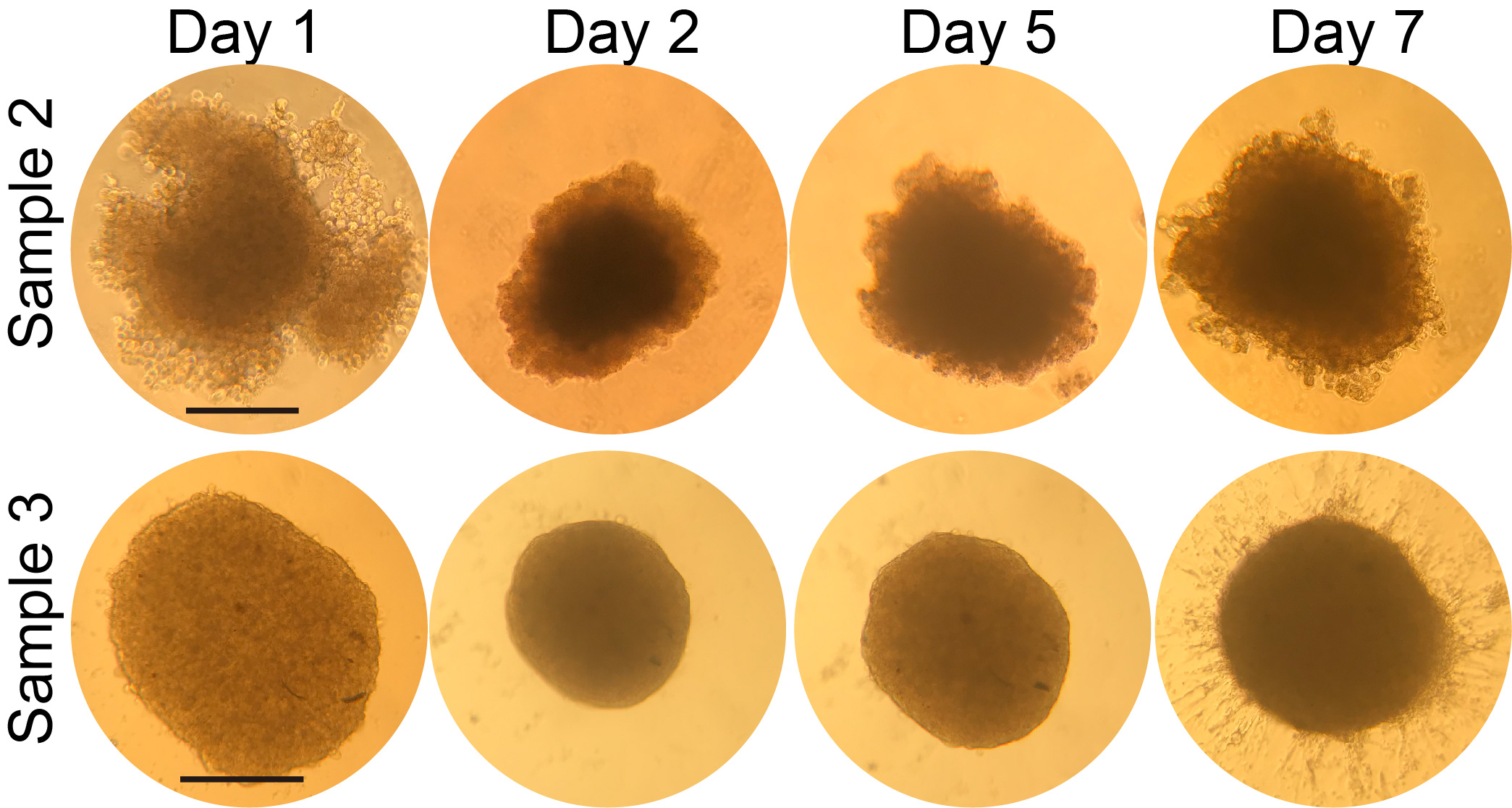

Supplement: Supplementary file 5 — Supporting Information [file CTM2-12-e814-s006.jpg]

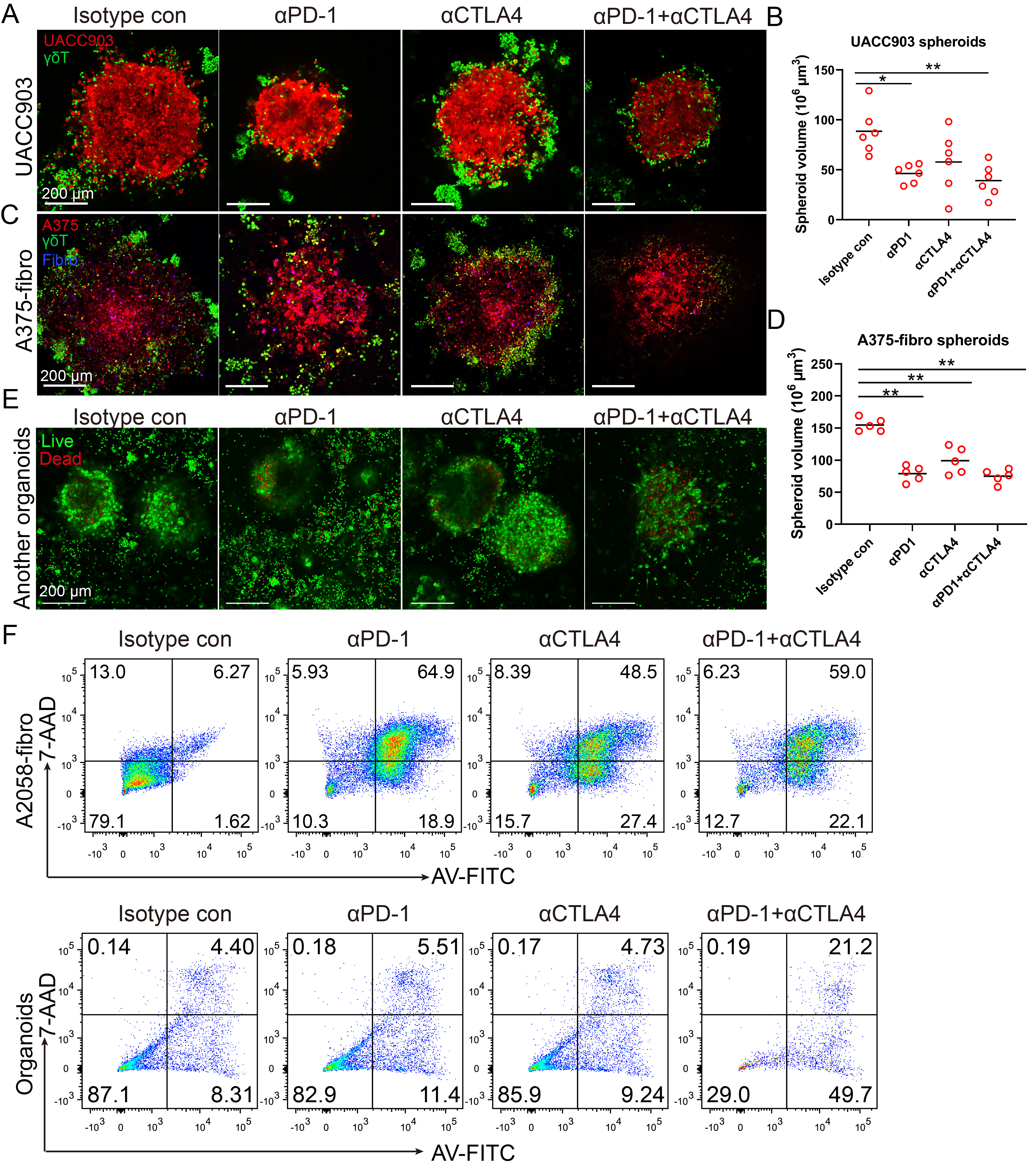

Supplement: Supplementary file 6 — Supporting Information [file CTM2-12-e814-s005.jpg]

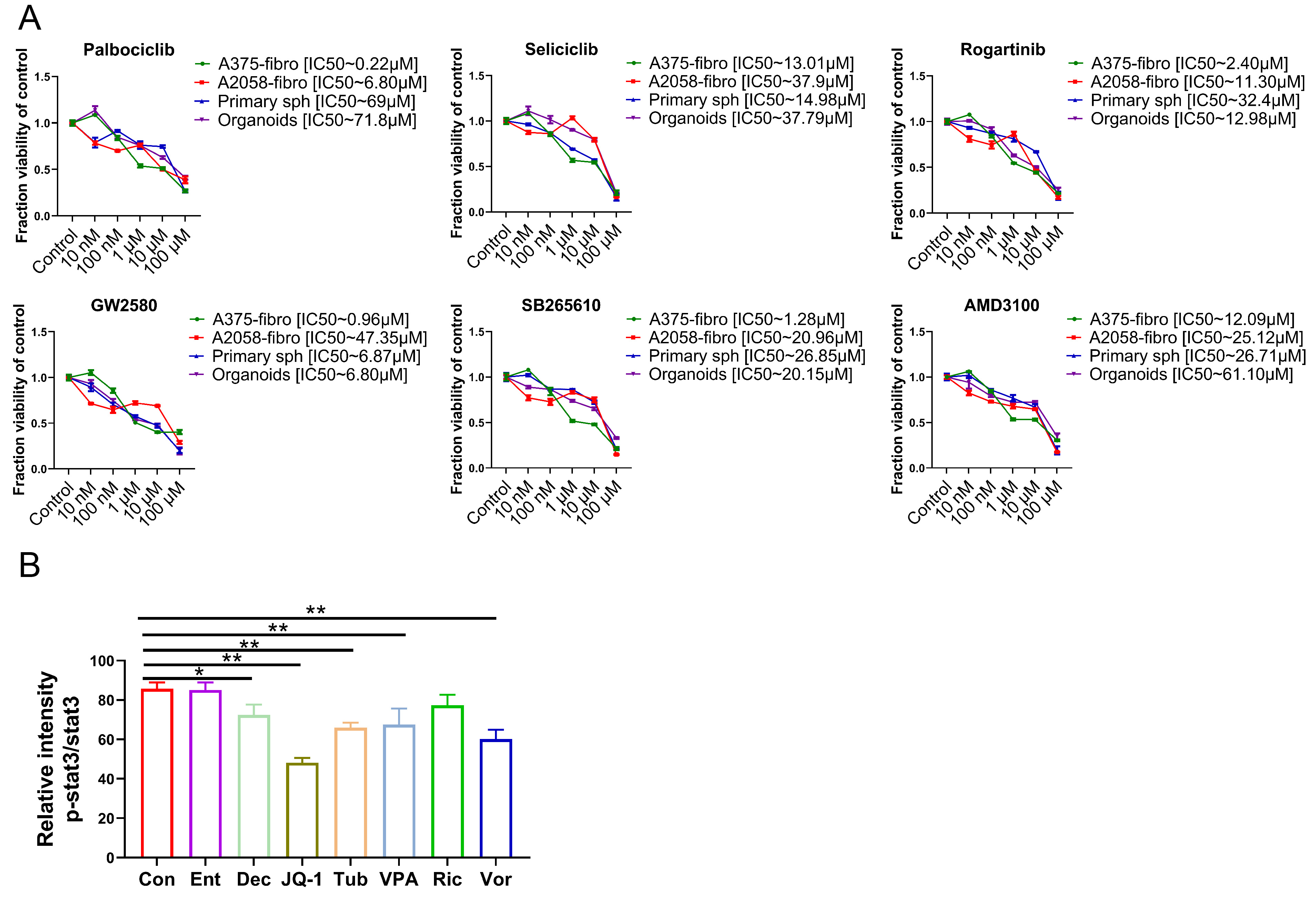

Supplement: Supplementary file 7 — Supporting Information [file CTM2-12-e814-s003.jpg]
